# Trophic ecology and nutritional status of northern shrimp in Canada’s sub-Arctic

**DOI:** 10.1371/journal.pone.0322745

**Published:** 2025-05-20

**Authors:** Gustavo Yunda-Guarin, Sheila Atchison, Krista D. Baker, Frédéric Cyr, Christopher C. Parrish, Wojciech Walkusz, Jonathan A. D. Fisher, Tyler D. Eddy

**Affiliations:** 1 Centre for Fisheries Ecosystems Research, Fisheries and Marine Institute, Memorial University, St. John’s, Newfoundland, Canada; 2 Freshwater Institute, Fisheries and Oceans Canada, Winnipeg, Manitoba, Canada; 3 Northwest Atlantic Fisheries Centre, Fisheries and Oceans Canada, St. John’s, Newfoundland, Canada; 4 Department of Ocean Sciences, Memorial University, St. John’s, Newfoundland, Canada; University of Messina, ITALY

## Abstract

In the Northwest Atlantic Ocean, northern shrimp (*Pandalus borealis*) play key ecological roles as mid-trophic level consumers and as prey to higher-trophic level predators, including commercial fish species. However, the effects of changing environmental conditions and biological processes on trophic interactions in sub-Arctic ecosystems, particularly on lipid storage and nutrient transfer from intermediate to high trophic levels, remain unclear. Biochemical tracer methods (*i.e*., fatty acids and stable isotopes) were employed to study the trophic ecology and stage-specific nutritional condition of *P. borealis* across different spatial and seasonal scales. Trophic markers indicated significant contributions from both diatoms and zooplankton to the diet of *P. borealis* and highlighted the adaptability of this species to opportunistic feeding strategies based on sinking phytodetritus. Our results revealed a strong seasonality in the lipid composition of *P. borealis*, with lipid dynamics being highly influenced by environmental conditions and resource availability. The primary lipid classes in *P. borealis* were storage triacylglycerols, accounting for over 50% of lipids observed, followed by membrane phospholipids. Eggs from ovigerous females exhibited the highest concentrations of total lipids and essential fatty acids, such as omega-3 fatty acids, underscoring the important ecological role of eggs in sub-Arctic food webs by providing high-quality lipid sources. Additionally, our findings indicated an increase in the total lipid content of shrimp eggs from spring to summer, suggesting that the early stages of *P. borealis* are vulnerable to changes in the timing of seasonal primary production, when females store large reserves of energy-rich lipids. This study highlights the large seasonal and temporal variability in the nutritional status of *P. borealis* and underlines the importance of understanding lipid dynamics in assessing the resilience of populations to environmental changes.

## 1. Introduction

Northern shrimp (*Pandalus borealis*, Krøyer, 1838 [1]) populations are widespread in the North Atlantic Ocean and represent some of the most valuable fisheries in Eastern Canada [2]. This species plays essential ecological roles in key ecosystem processes, acting as both mid-trophic level consumers and prey to higher-trophic level predators such as seabirds, seals, whales, and important commercial fish species [3,4]. Through their feeding, shrimp contribute to bottom-up processes by recycling nutrients and supporting energy transfer from primary producers to higher trophic levels. Simultaneously, they are integral to top-down processes, as their populations may be regulated by predation from top predators, influencing trophic dynamics and shaping sub-Arctic food web structure. As opportunistic feeders, shrimp consume a diverse diet, including phytoplankton, detritus, crustaceans, molluscs, jellyfish, and plant material [5–7].

Despite the importance of this species in high-latitude food webs and lucrative commercial fisheries, their trophic ecology, lipid biochemistry, energetic adaptations and mechanisms influencing their population dynamics are still poorly understood. *Pandalus borealis* is a sequential protandric hermaphrodite species that usually changes sex from male to female between four to seven years of age [8]. In northern latitudes, each individual undergoes a developmental process in which they are born as a male, then transition to an intersexual phase, and ultimately mature into a female [9]. This sex change strategy, common to many crustaceans, allows individuals to grow and store energy as males before transitioning to females, providing more energy for egg production [10]. Colder waters in northern latitudes slow growth, delaying the sex change [11]. As a result, *P. borealis* populations are highly sensitive to seasonal and long-term changes in climate, which can affect their life cycle and reproductive success [9].

While annual scientific assessments of shrimp population dynamics and stock productivity occur within some management areas, accurately forecasting regional productivity and potential fisheries yields remains challenging, especially for shrimp stocks that have never been assessed [12]. This is largely due to an incomplete understanding of the mechanisms influencing population dynamics and a lack of detailed knowledge of their life history, especially the variables affecting growth and abundance in the early stages [13]. Environmental changes are expected to affect key aspects of shrimp life stages, including egg survival, embryonic development, larval characteristics, hatching, and recruitment [14–16]. Due to the complex annual life cycle of *P. borealis*, both localized and large-scale alterations in environmental variables, such as temperature and pH, are likely to impact the survival, abundance and distribution of this species in the North Atlantic Ocean [17–19]. Indeed, historical declines in *P. borealis* populations in the Northwest Atlantic Ocean have been linked to climate-driven increases in water temperature and shifts in predation pressure [20]. In addition, climate-induced environmental changes may affect the physiology and composition of aquatic organisms, altering fatty acid composition and impacting the transfer of essential lipids within food webs [21,22].

Lipids are critical throughout the life cycle of marine species, supporting important physiological processes such as growth and reproduction [23]. Storage lipids like wax esters and triacylglycerols (TAG) provide high-energy reserves, whereas structural lipids such as phospholipids (PL) serve as fundamental components of cell membranes [24]. At high latitudes, FAs are biosynthesized mainly by primary producers (*e.g*., ice-associated algae and phytoplankton) [25]. Ice algae, compared to phytoplankton, contain higher proportions of long-chain polyunsaturated fatty acids (PUFAs), making them a critical high-quality food source for herbivores and subsequent trophic levels [26]. Among PUFAs, arachidonic acid (20:4ω6; ARA), eicosapentaenoic acid (20:5ω3; EPA), and docosahexaenoic acid (22:6ω3; DHA) are essential fatty acids (EFAs) primarily synthesized by microalgae in aquatic environments [27]. Since marine invertebrates, including arthropods, can synthesize only limited amounts of EFAs *de novo*, they rely on their diet to obtain these important nutrients [23]. Thus, variations in dietary composition may directly influence the lipid profiles of marine invertebrates, further altering nutritional dynamics within food webs [21]. While marine invertebrates have lower levels of lipids compared to primary producers such as phytoplankton and ice algae, they remain an essential source of PUFAs in marine food webs, underscoring their crucial ecological role in supporting higher trophic levels [28].

To provide new insights into trophic interactions and nutrient transfer, trophic biomarker approaches such as SI and FAs have proven to be powerful tools, opening new research avenues in trophic ecological studies [29]. Stable isotopes of carbon (*δ*^13^C) and nitrogen (*δ*^15^N) provide time- and space-integrated insights into species’ diets and habitat use, helping to quantify aspects of the food web [30]. Nitrogen isotope ratios, which show a consistent enrichment of approximately 2.3‰ per trophic level in aquatic environments, are used to estimate the trophic positions of consumers [31]. Carbon isotope ratios, with an enrichment of 0–2‰ per trophic level, help determine the reliance of consumers on different food sources [32]. Besides, FAs are exclusively biosynthesized by particular primary producers and integrated into marine species with little or no modification of the original structure, making them valuable as biomarkers of dietary sources [25,33,34]. Using a multi-dietary tracer approach that included both trophic biomarkers (*i.e*., stable isotopes and fatty acids) the main objective of this study was to provide new insights into the foraging ecology and nutritional status of *P. borealis*. The following research questions are addressed in this paper: **Q**_**1**_. How do variations in habitat and seasonal changes in food availability influence the diet composition and feeding strategies of *P. borealis*? **Q**_**2**_. What are the spatial and temporal patterns in fatty acid profiles and lipid storage across different maturity stages of *P. borealis*, and how do shifts in diet or prey availability impact their nutritional conditions? **Q**_**3**_. How does combining multi-dietary tracer approaches enhance our understanding of *P. borealis* ecology and establish a baseline for evaluating the future status of shrimp conditions in a changing environment?

## 2. Materials and methods

### 2.1. Study area

**Fig 1:**
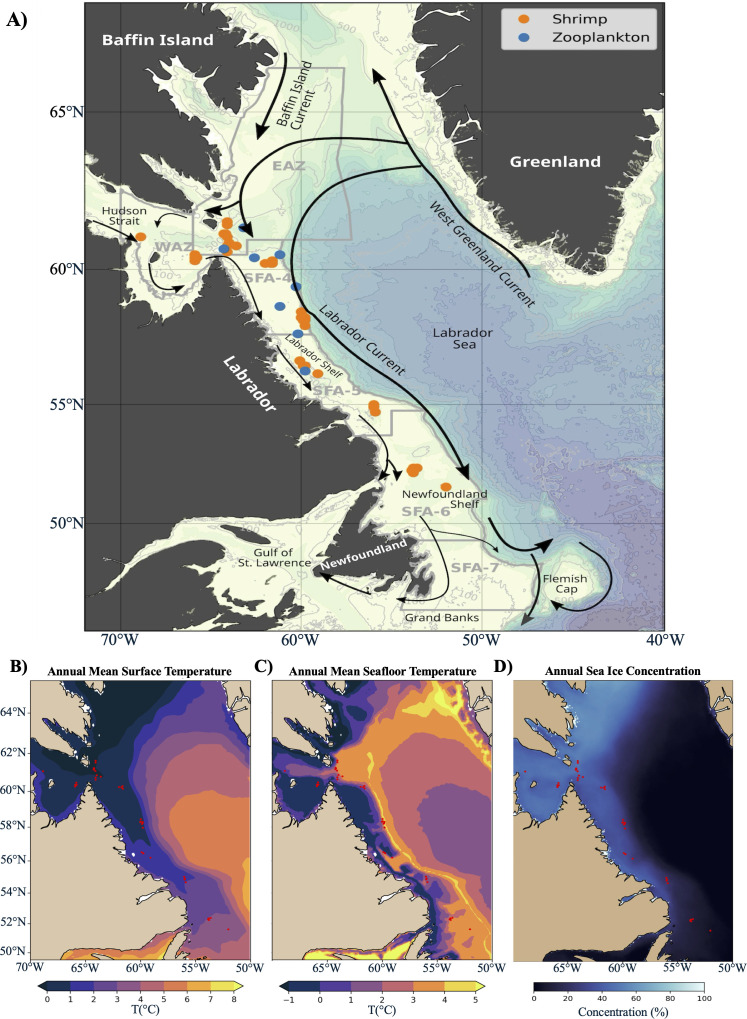
Maps of the study area. A) The location of northern shrimp (*Pandalus borealis*) and zooplankton sampling sites respective to shrimp fishing areas (SFAs; gray polygons). The Eastern Assessment Zone (EAZ) and Western Assessment Zone (WAZ) correspond to SFA2 and SFA3, respectively. The main bathymetric features, shown in color, have been extracted from GEBCO Compilation Group (2023). Schematic surface flow patterns (arrows), together with the main currents and topographic features, are indicated in black. Orange points represent sites where *P. borealis* was sampled between 2022 and 2023 across five shrimp fishing areas (SFAs 4-6, WAZ and EAZ). Blue points represent sites where zooplankton was sampled in 2023 across eight stations (from north to south: Hatton, Killinek Main, Isecold-3, Hatton Basin, SagBank, Isecold-2, Isecold-1, and Sentinel). The annual mean surface (B) and bottom (C) temperatures averaged over the sampling years (*i.e*., 2022-2023) have been extracted from the Global Ocean Physics Analysis and Forecast (GLORYS). D) Annual mean sea ice concentration (2022-2023) extracted from the MASAM2 dataset for the region of interest [43].

The study region encompasses the lower Arctic and sub-Arctic, extending from the Labrador Shelf in the south to Hudson Strait and the southern Davis Strait in the north (Fig 1). This area covers five shrimp fishing areas (SFAs) designated for the biomass assessment and resource management of shrimp stocks. The northern part of this region is characterized by intense mixing and transformation of water masses [35]. Further south, the Labrador Current interacts with warmer Atlantic waters from the West Greenland Current and colder Arctic waters from the Baffin Island Current and Hudson Strait outflow, creating a dynamic system of water exchange [36] (Fig 1A). The hydrodynamics of the Labrador Shelf are primarily driven by this southward-flowing Labrador Current system, which consists of a narrow, cold Arctic-origin coastal current on the shelf and a wider main branch carrying subpolar waters along the slope [37]. The latter is an essential component of the anticlockwise subpolar gyre that largely contributes to the climate variability of the North Atlantic [38]. Freshwater runoff and sea-ice melt significantly influence interannual variation in salinity and the strength of the Labrador Current [39]. These currents establish a larval connectivity system critical for determining *P. borealis* settlement densities in the region [40]. Interannual variability in sea ice extent along the Labrador Shelf is primarily driven by fall and winter air temperatures and snow conditions [41], with greater ice extent and colder spring air temperatures linked to colder waters, whereas reduced ice extent and higher spring surface salinity are linked to warmer winter air temperatures [42].

### 2.2. Sample collection

Sample collection of *Pandalus borealis* was conducted with vessels affiliated with the Canadian Association of Prawn Producers (CAPP) and the Northern Coalition (NC) across five Shrimp Fishing Areas (SFAs) (Fig 1A; S1 Table). To determine fatty acid profiles for lower and middle trophic levels, meso- and macro-zooplankton samples were collected during the summer of 2023 onboard the Canadian research icebreaker *CCGS Amundsen* (Fig 1A; S1 Table). Sampling was conducted using a double square net (DSN) with a mesh size of 500 µm, covering a 1m^2^ collection area, deployed to a maximum depth of 100 meters of the water column. After collection, all samples were immediately frozen at –20°C for further biochemical analyses. Shrimp samples were categorized by sex and reproductive stages (S1 Table). Total weight and morphometric measurements (*i.e.,* total body length and carapace length) of each individual were recorded following shellfish multi-species survey protocols established by Fisheries and Oceans Canada (S1 Table). Zooplankton samples were frozen as bulk, with a subsample taken for further taxonomic composition determination. The taxonomic analysis was performed to the lowest possible level (data, not included in this paper), which has allowed for analysis of the relative contributions of zooplankton types based on their feeding modes.

### 2.3. Stable isotope analyses

In total, 500 individual shrimp samples were used for stable isotope analyses. Muscle tissues from shrimp samples were freeze-dried at –50°C, ground into a fine powder using a mortar and pestle, and thoroughly cleaned between samples to prevent cross-contamination. Tools were rinsed with 95% ethanol and wiped clean after processing each sample. Stable nitrogen and carbon isotope ratios were measured using a Carlos Erba Elemental Analyser coupled to a Thermo DeltaV isotope-ratio mass spectrometer in the Stable Isotope Laboratory of Memorial University, St. John’s, Canada. Replicated measurements of international standards (USGS40 and USGS41 from the International Atomic Energy Agency; B2151 from Elemental Microanalysis) established measurement errors of ≤ 0.13‰ for *δ*^13^C and *δ*^15^N. All SI results are expressed in delta (*δ*) units (*δ*^13^C, *δ*^15^N) as the per mil (‰) difference with respect to standards: *δ*X (‰) = [(R_Sample_ – R_Standard_)/R_Standard_] × 10^3^, where X is ^13^C or ^15^N of the sample and R represents either ^13^C/^12^C or ^15^N/^14^N. Standards were calibrated against the international references Vienna PeeDee Belemnite (VPDB) for carbon and atmospheric air for nitrogen. Additionally, 24 bulk zooplankton samples were processed for SIA using the same procedures as described for shrimp. Analytical blanks and replicate analyses were included at regular intervals to ensure data quality. Zooplankton samples were initially separated by fishing areas to account for spatial variability in isotopic baselines; however, they were later grouped to ensure a sufficient representative number for trophic position analyses. Full SIA results are provided in supplementary material (S2 and S8 Tables).

### 2.4. Lipid class and fatty acid analyses

Over 900 individual samples, including *P. borealis* at various maturity stages, eggs from ovigerous females, and bulk zooplankton, were analyzed for FA composition, lipid classes, and total lipid content. To our knowledge, this is one of the most extensive studies of this type ever conducted on these taxa. The maturity stages of *P. borealis* were categorized into the following discrete classes based on sexual maturity: males, first-time spawning females with head roe, multiple-spawning females with head roe, multiple-spawning females without head roe, and ovigerous females (S1 Table). This classification was derived from the 2023 spring multi-species survey conducted by the Department of Fisheries and Oceans Canada (DFO, unpubl. data). Lipid analyses were conducted at the Department of Ocean Sciences, Memorial University of Newfoundland, St. John’s, Canada. Total lipids were extracted using a chloroform/methanol/water solution (2:1:1, v/v) following the established protocol of Folch et al. [44] as modified by Parrish [45]. Samples were homogenized, sonicated, and centrifuged three times for lipid extraction. The lipid extracts were stored at −20°C in 2 ml vials, capped under nitrogen and sealed with Teflon tape until further analysis. A three-step development system was used to separate and quantify lipid classes [46]. Lipid content and classes were quantified using a Chromarod-Iatroscan (Mark V) TLC/FID system. FAs in shrimp were transesterified from the lipid extracts and analyzed as methyl esters (FAME) using an HP6890 GC FID system equipped with a 7683 autosampler. Zooplankton lipid results are included in the supplementary material (S6 and S7 Tables).

To assess variations in the contribution of different food sources, multiple fatty acid markers were used as dietary indicators. Pelagic markers such as C16 FAs (*e.g*., 16:1ω7, 16:4ω1) and 20:5ω3 are primarily produced by ice-associated and pelagic diatoms, while 18:4ω3 and 22:6ω3 FAs are predominantly linked to dinoflagellates [47–49]. Long-chain C20 FAs (*e.g*., 20:1ω9, 20:1ω11) and C22 FAs (*e.g*., 22:1ω9, 22:1ω11) serve as reliable indicators of a diet rich in Calanus copepods [50,51]. Benthic and coastal markers included FAs associated with macroalgae (20:4ω6), vascular plants (18:3ω3, 18:2ω6), green macroalgae (18:2ω6), and bacteria (18:1ω7) [52]. Concerning lipid classes, a high amount of free fatty acids (FFA) indicates the occurrence of lipid hydrolysis and suggests that samples had undergone degradation processes either before, during, or after the storage period [53]. If hydrolysis has occurred, TAG content cannot reliably be used as a measure of condition due to the potential loss of some lipids [54]. Therefore, in this study, the relative proportion of FFA was used as an indicator of sample preservation quality, with samples exceeding 20.5% FFA of the total lipid content being excluded from lipid class analyses. In addition, the nutritional condition of *P. borealis* samples was assessed based on the following lipid indicators: (1) the amount (as mg/g WW) of total lipids content per sample; (2) the amount (as mg/g WW) of the lipid classes TAG and PL; (3) the sum (as % total lipid) of the EFAs: ARA: 20:4ω6, EPA: 20:5ω3, and DHA: 22:6ω3, and (4) TAG-sterol ratios as proposed by Fraser [55].

### 2.5. Trophic levels

The estimation of trophic levels was used to characterize the functional role of *P. borealis* individuals across SFAs. The trophic level of shrimp was assessed using the ‘OneBaseline’ model from the Bayesian tRophicPosition package in R (v.4.4.1) [56]. This model applies the equation: *δ*^15^*N*_c _= *δ*^15^N_b _+ Δ*N* (TL – λ). Where *δ*^15^*N*_c_ is the nitrogen stable isotope value of the consumer (shrimp) for which the trophic level is estimated, *δ*^15^*N*_b_ represents the nitrogen isotope ratio of a known trophic level (herbivorous zooplankton), Δ*N* is the trophic discrimination factor (TDF) for nitrogen, and λ is the trophic level of baseline sources. In this study, λ was set to 1.0, representing basal primary producers. Trophic discrimination factors (Δ*N* and Δ*C*) were sourced from McCutchan et al. [57], with values of 2.9 ± 0.32 (SE) for nitrogen and 1.3 ± 0.3 (SE) for carbon. The trophic levels were categorized as: low trophic level (≤ 2) indicates primary consumers, such as herbivores and filter feeders; intermediate trophic level (> 2 and < 3) represents secondary consumers (*e.g*., omnivores); and high trophic level (≥ 3) indicates higher trophic level consumers, including scavengers/detritivores.

### 2.6. Environmental analyses

Sea bottom temperature and sea ice concentrations were evaluated as explanatory variables to explain variation in trophic biomarkers. Surface and seafloor water temperature were extracted from the European Union Copernicus Marine Service Information (CMEMS) Global Ocean Physics Analysis and Forecast (GLORYS), available at https://doi.org/10.48670/moi-00016 [consulted August 2nd, 2024]. Sea ice concentration data were extracted from the National Snow and Ice Data Center (NSIDC) Daily 4 km Arctic Sea Ice Concentration, Version 2 (MASAM II), accessible at https://doi.org/10.7265/bqd9-vm28 [consulted March 5th, 2024]. Annual averages (2022–2023) for surface and seafloor temperatures and sea ice concentrations are presented in Fig 1 (panels B to D, respectively). For each sampling site, we calculated the average sea ice concentration (SIC%) over the three months before sampling and the average bottom temperature (°C) for the month before the sampling date. This period was selected due to the isotopic and lipid turnover rates in the tissues of some high-latitude crustaceans ranging from less than one month to three months [58]. These turnover rates can significantly impact the isotopic and fatty acid composition of these consumers [58].

### 2.7. Statistical analyses

All statistical analyses were performed using R (v.4.4.1) (https://www.R-project.org/) in RStudio (v. 2024.04.2 + 764). Stable isotope and fatty acid values were compared across SFAs and seasons using a two-way analysis of variance (ANOVA), followed by Tukey’s post-hoc test for pairwise comparisons. Linear regression analysis was employed to develop a model that explains the relationship between a dependent and one or more independent variables. Specifically, these models were used to simultaneously evaluate the effects of shrimp sex, weight, and size, along with environmental variables such as depth, bottom temperature, and sea ice concentration, on SI and FA profiles. The normality of residuals was tested by examining the characteristic Quantile-Quantile (QQ) plot [59]. If residual normality and homoscedasticity assumptions were not met, response variables were log-transformed. Contrasts were estimated using ‘emmeans’ package (v.1.10.3) based on Searle et al. [60]. Model fit evaluations and assumption checks were done through visualizations using the ‘performance’ package (v.0.10.4) [61]. The isotopic niche space occupied by shrimp populations was calculated using the standard ellipse area, or ‘niche size’ method, available in the R ‘SIBER’ package [62]. Additionally, the probabilistic approach outlined by Jackson et al. [62] was applied to estimate the mode and credible intervals of the Bayesian-simulated Standard Ellipse Areas (SEAb). To ensure the robustness of our results, the sample size-corrected standard ellipse area (SEAc) approach was used to reduce the impact of extreme *δ*^13^C and *δ*^15^N values on the analysis. SEAc encompasses approximately 40% of the isotopic observations within each shrimp fishing area (SFA), making it less susceptible to the influence of sample size variations and isotopic outliers [62]. The visualization of FA profiles of shrimp was carried out using a principal component analysis (PCA). This method highlights the linear correlations among the variables and identifies the specific FAs that significantly contribute to distinguishing between different observed groups. FA that contributed less than 1.0% to the total FA profile on average were excluded from the analysis.

## 3. Results

### 3.1. Stable isotope composition

**Fig 2:**
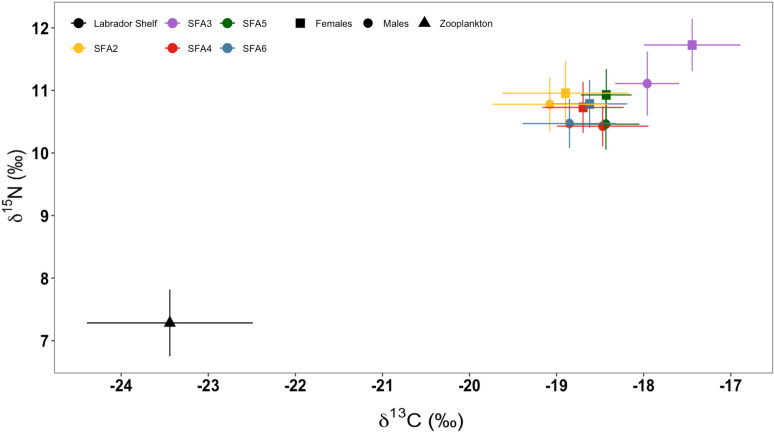
Carbon and nitrogen isotopic composition of northern shrimp (*Pandalus borealis*) and zooplankton. Stable isotope biplots illustrate the isotopic composition of *Pandalus borealis* across the shrimp fishing areas SFA2 (yellow), SFA3 (purple), SFA4 (red), SFA5 (green), and SFA6 (blue). The isotopic composition is represented by solid symbols: squares for *P. borealis* females, dots for males, and a triangle for zooplankton. Each shrimp data point is the mean for the group with error bars representing ± SD. Sample sizes are presented in S2 and S7 Tables.

*Pandalus borealis* exhibited a relatively wide range of isotopic compositions across different shrimp fishing areas (SFAs; Fig 2). The stable isotopic composition (*δ*^13^C and *δ*^*15*^*N*) of *P. borealis* varied significantly among SFAs, seasons, and maturity stages (p < 0.001). On average, *δ*^13^C values in the muscle of females varied from −17.4 ± 0.6 ‰ to −18.9 ± 0.7 ‰ (mean ± SD), while *δ*^13^C values in the muscle of males ranged from −18.0 ± 0.4 ‰ to −19.1 ± 0.7 ‰ (S2 Table). Among SFAs, the most ^13^C-enriched value (*δ*^13^C = −16.7 ‰) was recorded in female shrimp in SFA3, whereas the most ^13^C-depleted value (*δ*^13^C = −21.2 ‰) was observed in male shrimp in SFA2, both in autumn.

Stable isotope analysis also revealed that male shrimp in SFA6 had the most ^15^N-depleted values (9.5 ‰) in winter. In contrast, the most ^15^N-enriched value (12.4 ‰) was recorded for female shrimp in SFA3 in autumn (Fig 2). The average *δ*^15^N values in females ranged from 10.7 ± 0.4 ‰ to 11.7 ± 0.4 ‰, whereas *δ*^15^N values in males ranged from 10.5 ± 0.4 ‰ to 11.1 ± 0.5 ‰ (S2 Table). Linear models indicated that environmental conditions may influence fluctuations in the isotopic composition of *P. borealis*, with a significant interaction effect between sea-ice concentrations (p < 0.01) and temperature (p < 0.001) on *δ*^13^C isotopic values (S3 Table). Additionally, this analysis denoted a significant effect of depth, sea-ice concentrations, and bottom temperature on *δ*^15^N signatures (p < 0.001). The isotopic composition of bulk zooplankton, used as a benchmark to further trophic position discussion, showed it has much lower values of *δ*^15^N than the shrimp (Fig 2; S8 Table). The average *δ*^15^N value was 7.3 ± 0.5 ‰, while the average value of the *δ*^13^C was −23.4 ± 0.9 ‰.

### 3.2. Trophic positions occupied by northern shrimp

**Fig 3:**
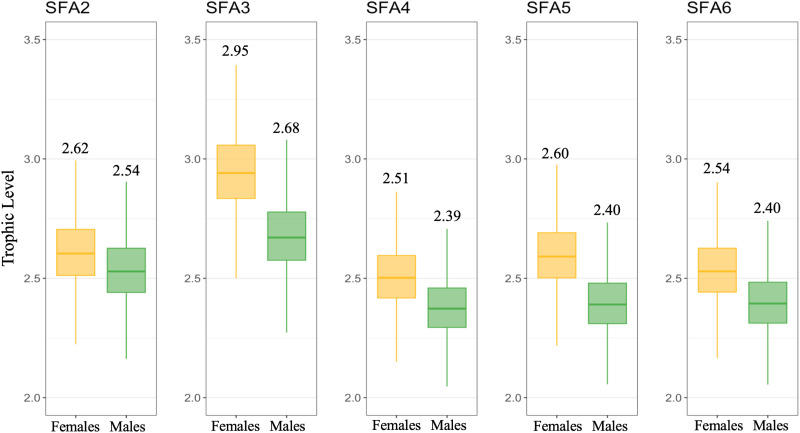
The estimated trophic levels of female and male northern shrimp (*Pandalus borealis*) in five shrimp fishing areas in Canada’s sub-Arctic. Horizontal lines represent the trophic levels occupied by different shrimp individuals (mean trophic level values given above the boxes; black numbers). The middle part of the boxes represents the interquartile range, *i.e.,* the middle quartiles (or the 75^th^ minus the 25^th^ percentile). The whiskers represent the variability outside the 75^th^ and 25^th^ percentile. Estimates were made using the ‘tRophicPosition’ model.

The taxonomic analysis of bulk zooplankton (not detailed here) revealed that the vast majority of the biomass consisted of the herbivorous *Calanus* species (*C. finmarchicus*, *C. glacialis,* and *C. hyperboreus*). In most samples, these species made up approx. 80% of the content, making this bulk-content analysis a justifiable benchmark approach. The trophic levels occupied by *P. borealis* ranged between the second and third trophic levels (Fig 3). Across the SFAs, females of shrimp consistently exhibited higher trophic levels compared to males (Figs 3 and S1). Notably, in SFA3, females displayed the highest trophic levels (mean = 2.95). In contrast, males in SFA4 showed the lowest trophic levels (mean = 2.39) in this study.

### 3.3. Isotopic niche dynamics of northern shrimp populations across spatiotemporal scales

**Fig 4:**
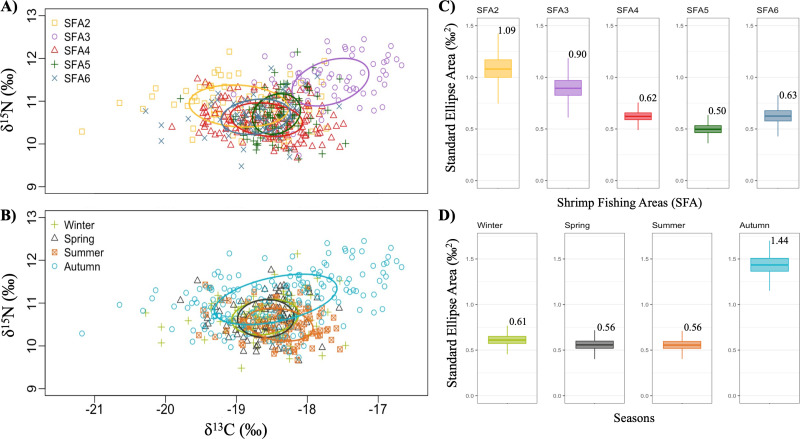
Stable isotope biplots illustrating seasonal and spatial changes in the isotopic niche structure of the northern shrimp (*Pandalus borealis*) population in Canada’s sub-Arctic. The positions occupied by females and males of shrimp in the isotopic space are represented by dots in each *δ*^13^C − *δ*^15^N biplot. Standard ellipses (solid lines) enclose the size-corrected standard ellipse area (SEAc, fits 40% of the data) of shrimp along the shrimp fishing areas **(A)**: SFA2 (yellow), SFA3 (purple), SFA4 (red), SFA5 (dark green), and SFA6 (dark blue); and seasons **(B)**: winter (light green), spring (dark grey), summer (orange), and autumn (light blue). Variation in the sizes of the standard ellipse areas (Figs C and D; with mean SEAc values given above the boxes) was calculated using SIBER.

The isotopic niches of *P. borealis*, measured as the size-corrected standard ellipse area (SEAc), varied across SFAs and seasons (Fig 4A and 4B). SEAc values ranged from 0.50 to 1.09 among SFAs and from 0.56 to 1.44 among seasons (Fig 4C and 4D). Variation in the isotopic niche breadth along SFAs revealed a decrease in niche size from northern to southern regions (Fig 4C) and an increase in the isotopic niche breadth in autumn (SEAc = 1.44) compared to other seasons (Fig 4D). Based on δ^13^C *vs* δ^15^N biplots, the most significant isotopic differences along the carbon axis were observed between the shrimp populations SFA2 and SFA3, despite their geographical proximity, as well as the absence of niche overlap between SFA3 and the other SFAs (Figs 1 and 4A). In contrast, higher overlaps were observed between the niches of SFAs 4, 5, and 6 (Fig 4A) and the spring and winter seasons (Fig 4B).

### 3.4. Fatty acid composition in northern shrimp

A total of 68 different FAs were identified in *P. borealis* tissues (*i.e.,* muscle and eggs). The relative abundances of these FAs varied among sex, tissues, seasons and fishing areas (Tables 1 and 2). Fourteen of these FAs had proportions exceeding 1% on average, collectively accounting for 89.7% of the total FAs identified in *P. borealis* (Tables 1 and 2). The overall lipid profile of *P. borealis* was dominated by the FAs: palmitoleic acid (16:1ω7; 13.8 ± 6.4%), eicosapentaenoic acid (EPA; 20:5ω3; 13.0 ± 5.0%), palmitic acid (16:0; 12.8 ± 3.1%), oleic acid (18:1ω9; 11.4 ± 2.4%), docosahexaenoic acid (DHA; 22:6ω3; 8.9 ± 3.8%), docosenoic acid (22:1ω11; 6.1 ± 5.2%), vaccenic acid (18:1ω7; 6.1 ± 2.1%), and eicosenoic acid (20:1ω9; 5.9 ± 3.7%). Each of these eight FAs exceeded 5% of the total FAs in the shrimp profile. Despite the overall similarity in dominance, individual proportions of these FAs fluctuated throughout maturity stages, tissues, and spatiotemporal gradients (Tables 1 and 2; S2 Fig). For example, in SFA3, the highest proportions of 16:1ω7 were found in both female and male shrimp (mean ± SD: 23.8 ± 6.0% and 22.0 ± 5.8%, respectively) compared to other SFAs. In contrast, among seasons, a higher proportion of 16:1ω7 (16.7 ± 2.9%) was detected in shrimp eggs during the winter period. Similarly, variation in the relative contribution of EPA and DHA was observed, with higher proportions of EPA and DHA found in ovigerous female eggs during the spring and increases in the relative abundance of EPA and DHA recorded in eggs and females in areas SFA2, SFA4, and SFA6.

**Table 1:** Summary of individual lipids as a percentage of total FAs (mean ± SD) in *Pandalus borealis* tissues (muscle and eggs) collected across shrimp fishing areas (SFAs) in Canada’s sub-Arctic.

Fatty acids	Males (n = 74)	Females (n = 91)	Eggs (n = 18)	Males (n = 69)	Females (n = 70)	Eggs (n = 17)	Males (n = 121)	Females (n = 121)	Eggs (n = 17)	Males (n = 82)	Females (n = 89)	Eggs (n = 15)	Males (n = 50)	Females (n = 57)	Eggs (n = 15)
**SFA2**			**SFA3**			**SFA4**			**SFA5**			**SFA6**		
**Saturates (%)**															
14:0	3.4 ± 0.7	2.9 ± 0.7	3.5 ± 0.4	3.6 ± 1.0	3.5 ± 0.9	2.7 ± 0.4	**4.2 ± 1.3**	3.1 ± 1.0	3.6 ± 0.5	3.6 ± 0.8	3.3 ± 1.0	3.5 ± 0.4	3.9 ± 0.8	3.6 ± 1.1	3.3 ± 0.6
16:0	11.1 ± 3.2	12.6 ± 4.0	11.7 ± 0.6	13.1 ± 2.0	13.6 ± 2.3	12.2 ± 3.2	13.1 ± 3.2	13.6 ± 3.0	12.4 ± 1.1	**13.8 ± 2.9**	13.0 ± 3.9	13.4 ± 1.0	11.7 ± 3.1	11.6 ± 3.0	13.2 ± 1.1
18:0	1.6 ± 0.3	1.7 ± 0.4	0.9 ± 0.1	2.0 ± 0.5	2.2 ± 0.6	1.1 ± 0.1	2.2 ± 0.7	2.0 ± 0.5	1.1 ± 0.2	**2.3 ± 0.4**	2.2 ± 0.4	1.4 ± 0.2	1.7 ± 0.4	2.0 ± 1.4	1.4 ± 0.3
ΣSAFA	16.9 ± 3.2	18.0 ± 4.1	16.7 ± 0.6	19.5 ± 2.6	20.2 ± 3.0	16.7 ± 3.2	20.5 ± 3.8	19.7 ± 3.1	17.8 ± 1.3	**20.6 ± 2.7**	19.5 ± 4.1	18.9 ± 0.9	18.2 ± 3.3	18.0 ± 3.2	18.6 ± 1.1
**Monounsaturates (%)**															
16:1ω7	8.6 ± 4.4	9.3 ± 4.5	12.2 ± 2.3	22.0 ± 5.8	**23.8 ± 6.0**	19.3 ± 3.1	15.0 ± 5.9	11.4 ± 4.0	13.4 ± 2.2	13.7 ± 4.3	13.0 ± 5.0	17.7 ± 3.3	11.0 ± 2.7	10.7 ± 2.9	14.3 ± 2.9
18:1ω7	4.2 ± 1.6	5.0 ± 2.2	4.7 ± 1.0	8.0 ± 1.3	8.8 ± 1.7	**9.0 ± 2.1**	5.2 ± 1.4	5.5 ± 1.6	5.2 ± 1.4	6.9 ± 1.3	7.0 ± 2.1	7.0 ± 1.0	5.0 ± 1.5	5.2 ± 1.5	6.9 ± 1.4
18:1ω9	12.1 ± 2.1	12.7 ± 2.1	**15.3 ± 1.4**	9.1 ± 1.7	9.6 ± 2.5	11.2 ± 3.3	10.6 ± 2.6	12.3 ± 2.0	13.3 ± 2.2	10.7 ± 2.1	11.2 ± 1.8	11.1 ± 1.4	11.9 ± 1.4	12.0 ± 2.0	11.0 ± 1.8
20:1ω7	**2.2 ± 0.9**	1.9 ± 1.3	1.2 ± 0.3	1.7 ± 0.6	1.9 ± 0.6	0.9 ± 0.2	1.8 ± 0.8	1.7 ± 1.1	1.1 ± 0.2	1.5 ± 0.7	1.7 ± 1.1	0.9 ± 0.1	1.7 ± 0.6	2.0 ± 0.9	0.9 ± 0.1
20:1ω9	8.9 ± 3.8	7.3 ± 4.7	5.8 ± 1.3	3.2 ± 2.0	3.6 ± 2.5	2.8 ± 1.4	5.8 ± 2.7	5.5 ± 3.2	4.4 ± 1.1	4.7 ± 2.4	5.1 ± 2.9	3.1 ± 0.6	**9.8 ± 4.1**	9.4 ± 3.4	3.1 ± 0.8
20:1ω11	1.1 ± 0.6	0.9 ± 0.6	0.8 ± 0.2	1.1 ± 0.5	1.1 ± 0.8	0.7 ± 0.3	0.9 ± 0.4	0.7 ± 0.4	0.6 ± 0.2	1.0 ± 0.5	1.0 ± 0.4	0.6 ± 0.1	**1.3 ± 0.5**	1.2 ± 0.4	0.6 ± 0.1
22:1ω9	**3.4 ± 1.9**	2.3 ± 2.1	1.1 ± 0.2	0.9 ± 0.6	0.9 ± 0.6	0.5 ± 0.2	2.0 ± 1.5	2.2 ± 3.5	1.0 ± 0.3	1.2 ± 0.9	1.4 ± 1.1	0.6 ± 0.2	2.4 ± 1.3	2.6 ± 1.5	0.7 ± 0.3
22:1ω11	10.4 ± 5.8	8.2 ± 7.5	4.5 ± 1.2	2.9 ± 1.9	3.0 ± 2.5	1.6 ± 0.8	5.9 ± 3.6	5.2 ± 4.5	3.7 ± 1.0	5.0 ± 3.5	5.0 ± 3.8	2.4 ± 0.6	**10.7 ± 5.2**	**10.7 ± 5.8**	2.7 ± 0.8
ΣMUFA	54.6 ± 11.5	50.4 ± 13.8	47.7 ± 2.7	51.7 ± 6.1	55.4 ± 6.2	47.9 ± 1.5	49.2 ± 9.0	46.8 ± 10.5	44.6 ± 3.1	47.3 ± 9.9	48.0 ± 10.7	45.2 ± 2.3	56.8 ± 11.4	**57.0 ± 11.8**	42.5 ± 2.0
**Polyunsaturates (%)**															
18:2ω6 (LIN)	1.3 ± 0.5	1.4 ± 0.6	1.6 ± 0.4	2.6 ± 0.5	2.7 ± 0.7	**3.0 ± 0.8**	1.8 ± 0.4	1.6 ± 0.4	2.0 ± 0.5	2.0 ± 0.4	2.1 ± 0.6	2.8 ± 0.5	1.4 ± 0.4	1.3 ± 0.3	1.9 ± 0.3
20:4ω6 (ARA)	0.6 ± 0.3	0.8 ± 0.5	0.8 ± 0.2	0.9 ± 0.5	0.7 ± 0.3	1.2 ± 0.3	0.8 ± 0.5	1.0 ± 0.6	1.0 ± 0.4	1.0 ± 0.4	1.1 ± 0.6	**1.3 ± 0.3**	0.6 ± 0.3	0.7 ± 0.4	1.2 ± 0.3
20:5ω3 (EPA)	11.6 ± 4.9	13.8 ± 6.1	17.4 ± 1.6	12.0 ± 2.9	10.2 ± 3.5	18.8 ± 1.1	12.6 ± 4.0	14.8 ± 5.0	18.3 ± 1.4	13.5 ± 4.5	13.3 ± 4.8	17.4 ± 1.5	9.4 ± 4.3	9.7 ± 5.1	**19.8 ± 1.0**
22:6ω3 (DHA)	9.9 ± 3.9	**10.3 ± 4.4**	9.7 ± 1.7	6.2 ± 2.5	4.6 ± 1.9	6.2 ± 0.9	8.8 ± 3.3	10.2 ± 3.8	**10.3 ± 1.3**	9.9 ± 3.6	9.7 ± 3.9	8.1 ± 1.5	8.8 ± 4.0	8.2 ± 4.0	10.1 ± 1.5
ΣPUFA	27.6 ± 8.7	30.6 ± 10.0	34.8 ± 2.5	27.7 ± 6.0	23.3 ± 5.4	34.2 ± 1.5	29.2 ± 6.4	32.4 ± 8.3	36.7 ± 2.3	30.9 ± 7.9	31.0 ± 8.6	34.8 ± 1.9	24.1 ± 8.5	24.0 ± 9.3	**37.9 ± 1.6**
Total	90.5	91.2	91.2	89.2	90.1	91.1	90.5	90.9	**91.4**	90.8	90.1	91.0	91.3	90.9	91.0
Bacterial	1.4 ± 0.2	1.5 ± 0.4	1.2 ± 0.1	1.8 ± 0.8	1.9 ± 1.6	2.0 ± 3.2	1.8 ± 0.3	1.7 ± 0.5	1.4 ± 0.3	2.0 ± 0.7	**2.4 ± 2.5**	1.7 ± 0.3	1.5 ± 0.4	1.6 ± 0.4	1.7 ± 0.3
P/S	1.6 ± 0.3	1.7 ± 0.3	2.1 ± 0.2	1.5 ± 0.4	1.2 ± 0.4	**2.3 ± 1.3**	1.4 ± 0.3	1.6 ± 0.3	2.1 ± 0.2	1.5 ± 0.3	1.7 ± 1.1	1.8 ± 0.1	1.3 ± 0.3	1.3 ± 0.4	2.0 ± 0.1
Σω3	24.0 ± 8.4	26.7 ± 9.7	30.7 ± 2.8	20.7 ± 5.9	16.9 ± 5.4	27.8 ± 1.8	24.0 ± 6.6	27.7 ± 8.2	32.0 ± 2.2	25.6 ± 7.8	25.4 ± 8.5	28.5 ± 2.8	20.3 ± 8.2	20.1 ± 9.0	**32.7 ± 1.8**
Σω6	3.1 ± 0.8	3.2 ± 1.2	3.4 ± 0.6	5.8 ± 1.0	5.7 ± 1.2	**5.9 ± 1.2**	4.4 ± 1.3	4.0 ± 1.3	4.0 ± 0.8	4.8 ± 0.9	5.0 ± 1.1	5.5 ± 1.0	3.2 ± 0.7	3.2 ± 0.8	4.3 ± 0.6
Σω6/Σω3	0.1 ± 0.04	0.1 ± 0.1	0.1 ± 0.03	0.3 ± 0.1	**0.4 ± 0.1**	0.2 ± 0.1	0.2 ± 0.1	0.2 ± 0.1	0.1 ± 0.03	0.2 ± 0.1	0.2 ± 0.1	0.2 ± 0.1	0.2 ± 0.05	0.2 ± 0.1	0.1 ± 0.02
DHA/EPA	0.9 ± 0.2	0.8 ± 0.2	0.6 ± 0.1	0.5 ± 0.1	0.5 ± 0.1	0.3 ± 0.05	0.7 ± 0.2	0.7 ± 0.2	0.6 ± 0.1	0.7 ± 0.1	0.7 ± 0.1	0.5 ± 0.1	**1.0 ± 0.1**	0.9 ± 0.1	0.5 ± 0.1
Zooplankton	**26.4 ± 12.1**	20.9 ± 15.5	13.5 ± 2.8	10.0 ± 4.6	10.7 ± 6.5	6.7 ± 2.7	16.5 ± 8.6	15.6 ± 10.6	11.0 ± 2.5	13.6 ± 7.6	14.5 ± 8.8	7.7 ± 1.3	26.2 ± 10.9	26.2 ± 11.9	8.2 ± 1.8
Vascular plants	1.6 ± 0.4	1.7 ± 0.5	2.1 ± 0.3	2.8 ± 0.6	2.9 ± 0.7	**3.1 ± 0.7**	2.1 ± 0.4	1.9 ± 0.4	2.4 ± 0.4	2.3 ± 0.4	2.4 ± 0.6	**3.1 ± 0.5**	1.7 ± 0.3	1.6 ± 0.3	2.3 ± 0.3
ΣEFA	22.1 ± 8.7	25.0 ± 10.2	27.9 ± 2.8	19.0 ± 5.6	15.4 ± 5.3	26.2 ± 1.6	22.1 ± 6.8	26.1 ± 8.7	29.6 ± 2.4	24.4 ± 8.1	24.2 ± 9.0	26.7 ± 2.7	18.8 ± 8.4	18.5 ± 9.4	**31.0 ± 2.0**

EPA: polyunsaturated eicosapentaenoic acid; DHA: polyunsaturated docosahexaenoic acid; SAFA: saturated FA; MUFA: monounsaturated FA; PUFA: polyunsaturated FA; ω3: omega-3; ω6: omega-6; DHA/EPA: dietary ratio and EFA: essential fatty acids (20:4ω6; 20:5ω3; 22:6ω3). Only those fatty acids ≥1% in at least one group are included in this Table. The maximum values obtained are shown in bold.

**Table 2:** Summary of individual lipids as a percentage of total FAs (mean ± SD) in *Pandalus borealis* tissues (muscle and eggs) collected across shrimp fishing areas in Canada’s sub-Arctic.

Fatty acids	Males (n = 142)	Females (n = 155)	Eggs (n = 42)	Males (n = 86)	Females (n = 98)	Eggs (n = 24)	Males (n = 64)	Females (n = 68)	Eggs (n = 10)	Males (n = 105)	Females (n = 107)	Eggs (n = 6)
**AUTUMN**			**WINTER**			**SPRING**			**SUMMER**		
**Saturates (%)**												
14:0	3.5 ± 0.9	3.1 ± 0.9	3.3 ± 0.6	3.7 ± 0.9	3.7 ± 1.1	3.5 ± 0.5	3.5 ± 0.7	2.9 ± 0.8	2.9 ± 0.4	**4.5 ± 1.1**	3.3 ± 0.8	3.4 ± 0.5
16:0	12.8 ± 3.2	13.8 ± 3.6	12.0 ± 2.1	13.2 ± 3.9	12.9 ± 3.1	12.9 ± 0.7	12.0 ± 2.4	11.0 ± 3.5	**14.3 ± 1.1**	12.6 ± 2.4	13.1 ± 2.7	11.8 ± 0.6
18:0	1.9 ± 0.5	2.0 ± 0.6	1.0 ± 0.2	2.1 ± 0.9	2.1 ± 0.4	1.3 ± 0.1	2.1 ± 0.4	**2.2 ± 1.3**	1.6 ± 0.3	2.0 ± 0.4	1.8 ± 0.4	0.9 ± 0.2
ΣSAFA	19.0 ± 3.6	19.9 ± 4.1	16.9 ± 2.2	**20.0 ± 4.1**	19.5 ± 2.8	18.4 ± 0.9	18.5 ± 3.0	17.0 ± 3.8	19.5 ± 1.4	19.9 ± 3.2	19.1 ± 2.9	16.8 ± 0.6
**Monounsaturates (%)**												
16:1ω7	14.6 ± 8.5	14.6 ± 9.2	15.6 ± 4.3	11.9 ± 3.6	12.5 ± 4.5	**16.7 ± 2.9**	12.8 ± 4.5	11.1 ± 4.1	12.3 ± 1.8	16.5 ± 5.3	13.2 ± 4.5	12.5 ± 1.9
18:1ω7	6.2 ± 2.1	6.6 ± 2.7	6.5 ± 2.6	5.7 ± 1.9	6.1 ± 1.8	6.5 ± 1.2	6.3 ± 1.6	6.6 ± 2.1	**7.7 ± 1.2**	5.1 ± 1.7	5.5 ± 2.0	5.0 ± 0.9
18:1ω9	11.0 ± 2.6	11.8 ± 2.8	13.2 ± 3.0	11.6 ± 1.7	11.4 ± 1.9	11.0 ± 1.7	11.5 ± 2.7	12.2 ± 1.9	11.7 ± 1.4	9.5 ± 1.8	11.3 ± 1.9	**15.0 ± 2.4**
20:1ω7	1.8 ± 0.9	1.8 ± 1.2	1.0 ± 0.2	1.7 ± 0.8	1.8 ± 1.0	0.9 ± 0.1	1.9 ± 0.7	**2.1 ± 1.2**	0.9 ± 0.2	1.8 ± 0.6	1.7 ± 0.9	1.2 ± 0.4
20:1ω9	5.5 ± 3.9	5.0 ± 4.0	4.2 ± 1.8	**7.3 ± 4.3**	7.2 ± 4.0	3.2 ± 0.8	6.4 ± 3.4	6.7 ± 3.7	3.3 ± 0.9	6.1 ± 3.0	6.0 ± 3.6	6.0 ± 1.5
20:1ω11	1.1 ± 0.6	0.9 ± 0.7	0.7 ± 0.2	1.1 ± 0.5	1.0 ± 0.4	0.6 ± 0.1	**1.2 ± 0.4**	1.1 ± 0.4	0.6 ± 0.1	0.8 ± 0.3	0.7 ± 0.4	0.9 ± 0.2
22:1ω9	1.9 ± 1.8	1.7 ± 2.8	0.9 ± 0.4	1.9 ± 1.3	2.0 ± 1.5	0.7 ± 0.3	1.9 ± 1.4	**2.3 ± 2.8**	0.6 ± 0.2	2.2 ± 1.6	1.8 ± 1.6	0.9 ± 0.3
22:1ω11	5.5 ± 5.0	4.7 ± 5.3	3.0 ± 1.5	**7.8 ± 5.6**	7.5 ± 5.6	2.7 ± 0.7	7.0 ± 4.1	7.3 ± 5.2	2.6 ± 0.9	7.0 ± 4.6	6.5 ± 5.9	5.1 ± 1.8
ΣMUFA	50.8 ± 10.9	49.9 ± 12.6	46.9 ± 2.3	51.6 ± 13.0	52.2 ± 12.4	44.4 ± 1.9	51.8 ± 8.2	**52.3 ± 10.0**	41.7 ± 3.5	51.1 ± 7.0	49.0 ± 9.9	48.6 ± 3.5
**Polyunsaturates (%)**												
18:2ω6 (LIN)	2.0 ± 0.8	2.0 ± 0.9	**2.3 ± 0.9**	1.8 ± 0.5	1.7 ± 0.5	**2.3 ± 0.6**	1.7 ± 0.5	1.8 ± 0.6	2.1 ± 0.4	1.7 ± 0.5	1.6 ± 0.6	1.5 ± 0.3
20:4ω6 (ARA)	0.9 ± 1.8	0.7 ± 0.3	1.0 ± 0.3	0.7 ± 0.5	0.8 ± 0.5	1.1 ± 0.3	1.0 ± 0.5	1.2 ± 0.7	**1.4 ± 0.3**	0.7 ± 0.4	1.0 ± 0.5	1.1 ± 0.5
20:5ω3 (EPA)	12.6 ± 4.6	13.0 ± 5.7	18.2 ± 1.3	11.4 ± 5.4	11.4 ± 5.4	18.6 ± 1.7	11.8 ± 3.7	12.4 ± 4.7	**19.1 ± 1.4**	12.1 ± 3.0	14.3 ± 5.0	16.7 ± 2.5
22:6ω3 (DHA)	8.8 ± 4.5	8.9 ± 5.4	8.6 ± 2.4	9.4 ± 4.3	9.0 ± 4.4	8.8 ± 1.6	9.1 ± 2.2	9.0 ± 3.0	**10.7 ± 1.6**	7.9 ± 2.1	8.9 ± 2.8	8.1 ± 0.8
ΣPUFA	29.2 ± 8.5	29.2 ± 10.1	35.2 ± 2.0	27.5 ± 9.8	27.3 ± 10.0	36.1 ± 2.2	28.4 ± 5.9	29.0 ± 8.0	**37.7 ± 2.3**	27.8 ± 4.9	30.7 ± 7.6	33.7 ± 3.4
Total	89.9	90.8	91.3	**92.2**	91.0	91.0	90.3	89.7	91.7	90.5	89.9	90.1
Bacterial	1.7 ± 0.6	1.6 ± 1.1	1.6 ± 2.0	1.6 ± 0.6	1.7 ± 0.4	1.6 ± 0.2	2.1 ± 0.6	**2.5 ± 2.8**	1.7 ± 0.3	1.7 ± 0.3	1.8 ± 0.5	1.5 ± 0.5
P/S	1.5 ± 0.4	1.5 ± 0.4	**2.2 ± 0.8**	1.4 ± 0.3	1.4 ± 0.3	2.0 ± 0.2	1.5 ± 0.2	1.9 ± 1.2	1.9 ± 0.1	1.4 ± 0.2	1.6 ± 0.3	2.0 ± 0.2
Σω3	23.9 ± 8.8	24.2 ± 10.6	29.9 ± 2.9	23.0 ± 9.5	22.7 ± 9.7	30.5 ± 3.0	23.2 ± 5.3	23.8 ± 7.4	**32.3 ± 2.5**	22.6 ± 4.6	26.0 ± 7.3	29.1 ± 2.5
Σω6	4.4 ± 1.5	4.3 ± 1.7	4.6 ± 1.5	4.0 ± 1.5	4.0 ± 1.2	**4.8 ± 1.0**	4.5 ± 1.2	4.6 ± 1.4	4.7 ± 0.6	4.3 ± 1.3	4.0 ± 1.3	3.8 ± 1.2
Σω6/Σω3	0.2 ± 0.1	0.2 ± 0.2	0.2 ± 0.1	0.2 ± 0.1	0.2 ± 0.1	0.2 ± 0.1	0.2 ± 0.1	0.2 ± 0.1	0.1 ± 0.03	0.2 ± 0.1	0.2 ± 0.1	0.1 ± 0.03
DHA/EPA	0.7 ± 0.2	0.7 ± 0.2	0.5 ± 0.1	**0.9 ± 0.1**	0.8 ± 0.2	0.5 ± 0.1	0.8 ± 0.2	0.7 ± 0.1	0.6 ± 0.1	0.7 ± 0.2	0.7 ± 0.2	0.5 ± 0.1
Zooplankton	16.0 ± 11.4	14.4 ± 12.5	9.9 ± 3.8	**19.9 ± 11.7**	19.8 ± 11.9	8.3 ± 1.7	18.7 ± 9.4	19.7 ± 11.1	8.1 ± 2.0	18.2 ± 9.8	16.9 ± 11.7	14.3 ± 3.4
Vascular plants	2.3 ± 0.7	2.3 ± 0.9	2.6 ± 0.7	2.0 ± 0.5	2.0 ± 0.5	**2.7 ± 0.6**	2.0 ± 0.5	2.0 ± 0.6	2.5 ± 0.4	2.1 ± 0.5	1.9 ± 0.5	2.0 ± 0.3
ΣEFA	22.3 ± 8.9	22.6 ± 10.8	27.7 ± 2.5	21.6 ± 9.7	21.2 ± 10.2	28.5 ± 2.8	21.9 ± 5.4	22.5 ± 7.9	**31.1 ± 3.0**	20.7 ± 4.8	24.3 ± 7.8	25.9 ± 2.8

EPA: polyunsaturated eicosapentaenoic acid; DHA: polyunsaturated docosahexaenoic acid; SAFA: saturated FA; MUFA: monounsaturated FA; PUFA: polyunsaturated FA; ω3: omega-3; ω6: omega-6, DHA/EPA: dietary ratio and EFA: essential fatty acids (20:4ω6; 20:5ω3; 22:6ω3). Only those fatty acids ≥1% in at least one group are included in this table. The maximum values obtained are shown in bold.

**Fig 5:**
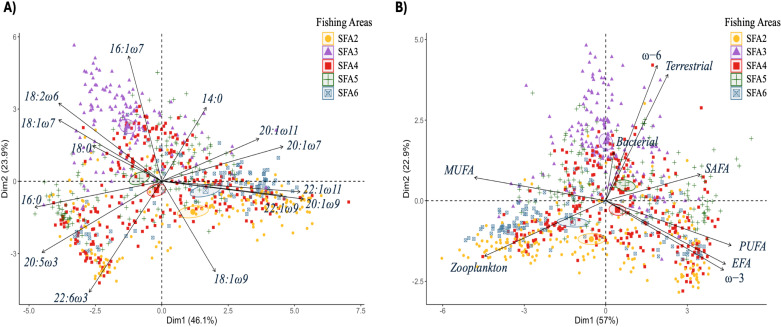
Principal component analysis (PCA) biplot illustrating the relative correlations between fatty acids found in northern shrimp (*Pandalus borealis*) and the fishing areas. Each point represents a shrimp sample from the following shrimp fishing areas: SFA2 (yellow), SFA3 (purple), SFA4 (red), SFA5 (dark green), and SFA6 (dark blue). The coloured ellipses represent 95% confidence intervals around the means of the sampled sites. Only fatty acids contributing ≥1% are included in Fig 5A. The correlation between the saturated fatty acids (SAFAs), monounsaturated fatty acids (MUFAs), and polyunsaturated fatty acids (PUFAs) and the fishing areas is shown in Fig 5B. The essential fatty acids (EFAs) are represented by omega-3 (ω3), omega-6 (ω6), and the sum of the EFAs 20:4ω6, 20:5ω3, and 22:6ω3.

The first two principal components of the PCA explained between 70% and 80% of the variation in FA composition among SFAs, indicating that shrimp individuals varied in lipid contributions across regions (Fig 5). For instance, PCA indicated that *P. borealis* in SFA3 showed significant contributions of the diatom-associated FA marker 16:1ω7 and the terrestrial-associated FA marker 18:2ω6 compared with other areas. In contrast, shrimp from other regions, such as SFA2, had high contributions of a mix of different FAs, including the diatom-associated FA marker 20:5ω3, the zooplankton-associated FAs 20:1ω9 and 22:1ω11, and the dinoflagellate-associated FA 22:6ω3 (Fig 5).

The relative abundances of SAFAs, MUFAs, and PUFAs were significantly different among SFAs (p < 0.001) and across maturity stages (p < 0.001) in this study. Females displayed distinct fatty acid profiles among SFAs, with SAFAs ranging from 18.0% in SFA6 to 20.2% in SFA3. MUFAs exhibited greater variability, from 46.8% in SFA4 to 57.0% in SFA6, while PUFAs ranged from 23.3% in SFA3 to 32.4% in SFA4. In males, SAFAs were slightly lower, ranging from 16.9% in SFA2 to 20.6% in SFA5. MUFAs among males fluctuated between 47.3% in SFA5 and 56.8% in SFA6, whereas PUFAs varied from 24.1% in SFA6 to 30.9% in SFA5. Shrimp eggs exhibited a distinct FA composition in SFA6, with the highest proportion of PUFAs (37.9 ± 1.6%) and the lowest proportion of MUFAs (42.5 ± 2.0%; Table 1). Linear model analyses indicated that both bottom depth (p = 0.001) and bottom temperature (p < 0.001) had a negative effect on SAFAs. Additionally, bottom depth had a positive effect on MUFAs (p < 0.01) but negatively affected PUFAs (p < 0.01). The weight of individual shrimp was also identified as an important variable influencing SAFAs, MUFAs, and PUFAs, with both SAFAs and PUFAs showing a negative correlation (p < 0.001 and p < 0.05, respectively) with shrimp weight in both females and males (S4 Table).

**Fig 6:**
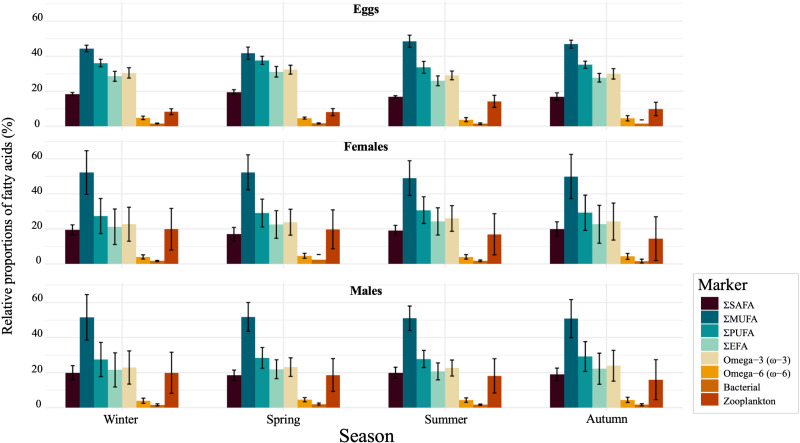
Bar plots illustrating the seasonal contributions of fatty acids in *Pandalus borealis* tissues. The bar plots represented the relative proportions of saturated FAs (SAFAs), monounsaturated FAs (MUFAs), and polyunsaturated FAs (PUFAs) found in shrimp tissues. The total contributions of omega-3, omega-6, and essential fatty acids (EFAs), as well as the contributions of FAs derived from bacterial and zooplankton sources, are also illustrated by the bars. EFAs are the sum of the fatty acids: 20:4ω6, 20:5ω3, and 22:6ω3.

Seasonal variation in the relative contribution of FAs was observed (Fig 6). For instance, male shrimp exhibited the highest contribution of SAFAs in wintertime (20.0 ± 4.1%, mean ± SD) (Table 2; Fig 6). A high contribution of MUFAs was observed in females of shrimp during spring (52.3 ± 10.0%). In contrast, a greater proportion of PUFAs was observed in the shrimp eggs during the same season (37.7 ± 2.3%). In this regard, in spring, an increase in the relative contribution of EFAs (*i.e*., 20:4ω6, 20:5ω3, and 22:6ω3) and omega-3 FAs was observed in eggs.

### 3.5. Lipid classes and nutritional condition of northern shrimp

Across fishing areas, the total lipid content (given as mg/g WW) in *P. borealis* varied from 16.2 ± 19.8 (mean ± SD, SFA5) to 34.1 ± 19.2 (SFA3). Overall, the main lipid classes in *P. borealis* tissues were dominated by triacylglycerols (TAG) accounting for more than 50% and phospholipids (PL) with more than 30% of the total lipid classes. Sterol content was generally found in low amounts in shrimp, with shrimp eggs displaying the highest values in the SFA2. Wax esters only occurred in trace amounts.

The proportions of TAGs and PLs varied across SFAs and maturity stages. TAG proportions (as a percentage of total lipids) were generally higher in SFA6, with females showing values ranging from 39.7 ± 28.5% in SFA2 to 69.4 ± 23.4% in SFA6. Similarly, TAGs in males varied from 48.9 ± 30.1% in SFA2 to 69.2 ± 19.8% in SFA6. In contrast, shrimp eggs exhibited lower variability in TAG proportions, ranging from 37.4 ± 12.6% in SFA6 to 53.8 ± 8.7% in SFA3. Phospholipid proportions showed an inverse trend in some cases, with females displaying the highest PL levels in SFA2 (32.2 ± 24.6%) and the lowest in SFA6 (13.8 ± 16.0%). Males exhibited PL values ranging from 16.5 ± 14.9% in SFA6 to 37.7 ± 22.5% in SFA3, while shrimp eggs had the most abundant PL proportions in SFA6 (50.0 ± 10.4%) and the least in SFA3 (39.9 ± 10.1%; Table 3). Linear models indicated that shrimp weight had a positive effect (p < 0.001) on TAG concentrations in shrimp tissues. In addition, shrimp weight (p < 0.001) showed a significant negative effect on PL levels (S5 Table). The environmental variables of sea ice concentrations and temperature had a significant influence on the proportions of TAG, PL, and total lipids in *P. borealis*.

**Table 3:** The relative concentration of lipid classes in northern shrimp (*Pandalus borealis*) throughout Canada’s sub-Arctic. The average lipid class contents (as mg/g WW) are measured in milligrams per gram of wet weight. The maximum values obtained are shown in bold.

Area^a^/ Season	Type	n^b^	Hydrocarbons	Steryl Esters/ Wax Esters	Ethyl Esters\ Methyl Esters	TAG^c^	FFA^d^	Alcohols	Sterols	AMPL^e^	PL^f^	Total Lipid content	TAG/ST^g^	EPA + DHA^h^
SFA2	Females	34	0.02 ± 0.1	0.4 ± 0.5	0.1 ± 0.2	9.9 ± 11.3	2.6 ± 2.9	0.02 ± 0.1	1.1 ± 0.7	0.4 ± 0.4	4.5 ± 4.9	19.0 ± 15.8	9.6 ± 10.5	1.4 ± 0.4
SFA2	Males	15	0.03 ± 0.04	0.3 ± 0.3	0.1 ± 0.1	9.9 ± 10.0	1.4 ± 0.7	0.02 ± 0.1	1.1 ± 0.3	0.4 ± 0.4	3.2 ± 2.1	16.5 ± 10.6	9.5 ± 7.8	1.2 ± 0.3
SFA2	Eggs	18	0.04 ± 0.1	0.5 ± 0.3	**0.4 ± 0.2**	34.9 ± 9.8	1.7 ± 0.7	0.01 ± 0.0	**1.9 ± 0.4**	1.0 ± 1.5	30.9 ± 10.7	71.4 ± 19.8	18.9 ± 4.9	1.8 ± 0.3
SFA3	Females	30	0.003 ± 0.01	0.1 ± 0.3	0.02 ± 0.05	13.3 ± 12.7	1.8 ± 2.7	0.002 ± 0.01	1.4 ± 2.0	0.7 ± 0.4	4.2 ± 3.4	21.5 ± 16.3	13.6 ± 12.8	2.1 ± 0.4
SFA3	Males	9	0.01 ± 0.01	0.03 ± 0.04	0.02 ± 0.02	7.8 ± 6.9	2.2 ± 1.6	**0.04 ± 0.1**	1.1 ± 0.5	0.7 ± 0.7	6.1 ± 5.6	18.0 ± 10.5	7.4 ± 5.7	2.2 ± 0.4
SFA3	Eggs	17	0.002 ± 0.004	0.3 ± 0.2	**0.4 ± 0.2**	**43.3 ± 8.2**	1.3 ± 0.8	0.0	1.4 ± 0.2	**1.6 ± 1.4**	**33.2 ± 13.7**	**81.6 ± 14.5**	30.8 ± 5.2	**3.1 ± 0.5**
SFA4	Females	29	0.03 ± 0.04	0.3 ± 0.6	0.1 ± 0.2	11.2 ± 14.7	1.5 ± 1.6	0.02 ± 0.1	1.1 ± 0.4	0.4 ± 0.4	3.8 ± 3.1	18.5 ± 16.0	12.2 ± 15.3	1.5 ± 0.4
SFA4	Males	17	0.03 ± 0.03	0.3 ± 0.4	0.2 ± 0.3	17.9 ± 11.5	2.5 ± 1.9	0.0	1.3 ± 0.4	0.7 ± 0.5	3.7 ± 1.6	26.5 ± 12.7	15.8 ± 12.9	1.1 ± 0.3
SFA4	Eggs	17	0.04 ± 0.04	0.3 ± 0.3	0.3 ± 0.2	23.7 ± 12.8	1.3 ± 0.4	0.0	1.5 ± 0.4	0.5 ± 0.4	22.0 ± 17.2	49.6 ± 24.2	16.1 ± 9.3	1.8 ± 0.2
SFA5	Females	18	0.02 ± 0.04	0.3 ± 0.5	0.06 ± 0.1	16.1 ± 15.6	1.6 ± 2.1	0.0	0.8 ± 0.4	0.4 ± 0.3	2.1 ± 1.5	21.3 ± 18.2	18.1 ± 14.2	1.6 ± 0.3
SFA5	Males	9	0.002 ± 0.005	0.2 ± 0.3	0.1 ± 0.1	16.5 ± 16.2	1.2 ± 0.3	0.0	0.9 ± 0.3	0.4 ± 0.3	2.4 ± 1.1	21.6 ± 17.6	17.0 ± 16.3	1.6 ± 0.4
SFA5	Eggs	16	0.03 ± 0.04	0.2 ± 0.1	0.3 ± 0.1	23.7 ± 10.9	1.8 ± 1.1	0.0	1.4 ± 0.3	0.5 ± 0.3	19.6 ± 8.0	47.5 ± 18.2	16.6 ± 7.3	2.2 ± 0.3
SFA6	Females	18	0.04 ± 0.1	0.6 ± 0.4	0.07 ± 0.1	29.3 ± 25.4	2.8 ± 3.4	0.01 ± 0.1	0.9 ± 0.2	0.4 ± 0.3	3.4 ± 4.4	37.5 ± 30.3	35.8 ± 29.3	1.1 ± 0.2
SFA6	Males	6	**0.06 ± 0.1**	**1.0 ± 0.9**	0.1 ± 0.1	39.1 ± 29.5	**3.5 ± 4.0**	0.0	1.1 ± 0.2	0.5 ± 0.2	4.6 ± 2.3	50.1 ± 31.0	**37.2 ± 30.8**	1.0 ± 0.2
SFA6	Eggs	14	0.01 ± 0.02	0.1 ± 0.1	0.2 ± 0.2	14.6 ± 8.2	1.9 ± 1.3	0.0	1.4 ± 0.3	0.4 ± 0.2	17.0 ± 5.9	35.6 ± 13.6	10.0 ± 5.0	2.0 ± 0.3
Autumn	Females	48	0.01 ± 0.02	0.2 ± 0.5	0.1 ± 0.2	11.1 ± 10.2	1.3 ± 1.3	0.01 ± 0.1	0.9 ± 0.4	0.6 ± 0.4	3.5 ± 3.9	17.7 ± 11.9	13.9 ± 11.7	1.7 ± 0.6
Autumn	Males	22	0.02 ± 0.03	0.2 ± 0.3	0.05 ± 0.1	11.7 ± 8.9	1.9 ± 1.3	0.01 ± 0.1	1.1 ± 0.4	0.7 ± 0.5	3.8 ± 4.1	19.6 ± 10.8	10.7 ± 6.5	1.5 ± 0.6
Autumn	Eggs	42	0.02 ± 0.04	0.3 ± 0.2	**0.4 ± 0.2**	36.1 ± 10.4	1.5 ± 0.6	0.004 ± 0.02	1.6 ± 0.3	1.0 ± 1.3	28.3 ± 8.9	69.3 ± 16.1	23.7 ± 7.7	**2.3 ± 0.7**
Winter	Females	24	**0.04 ± 0.1**	0.6 ± 0.5	0.1 ± 0.1	31.4 ± 22.1	**3.0 ± 3.4**	0.01 ± 0.1	1.0 ± 0.2	0.5 ± 0.2	3.3 ± 3.9	39.8 ± 26.3	34.9 ± 25.1	1.3 ± 0.3
Winter	Males	8	**0.04 ± 0.1**	**0.8 ± 0.9**	0.1 ± 0.1	**42.1 ± 21.6**	2.9 ± 3.6	0.0	1.0 ± 0.2	0.6 ± 0.2	2.7 ± 0.8	50.4 ± 24.9	**41.6 ± 22.3**	1.2 ± 0.2
Winter	Eggs	24	0.03 ± 0.03	0.2 ± 0.1	0.2 ± 0.1	23.1 ± 9.5	1.8 ± 1.3	0.001 ± 0.01	1.4 ± 0.3	0.5 ± 0.2	18.5 ± 6.4	45.8 ± 10.6	16.4 ± 7.7	2.1 ± 0.3
Spring	Females	20	0.02 ± 0.03	0.3 ± 0.4	0.1 ± 0.2	14.1 ± 15.2	1.4 ± 1.5	0.0	0.7 ± 0.2	0.5 ± 0.4	2.2 ± 1.1	19.3 ± 17.5	17.5 ± 17.2	1.3 ± 0.3
Spring	Males	12	0.02 ± 0.04	0.3 ± 0.3	0.2 ± 0.3	14.7 ± 11.7	1.3 ± 0.4	0.0	1.1 ± 0.5	0.5 ± 0.4	4.0 ± 2.5	22.2 ± 12.9	15.4 ± 14.7	1.4 ± 0.5
Spring	Eggs	10	0.03 ± 0.05	0.1 ± 0.2	0.1 ± 0.2	7.7 ± 11.0	1.6 ± 0.5	0.0	1.2 ± 0.3	0.4 ± 0.2	10.6 ± 8.9	21.8 ± 19.4	5.3 ± 5.2	1.8 ± 0.2
Summer	Females	37	0.02 ± 0.05	0.3 ± 0.5	0.04 ± 0.1	8.4 ± 12.7	2.7 ± 3.3	0.02 ± 0.05	1.6 ± 1.9	0.4 ± 0.4	5.3 ± 4.1	18.7 ± 18.3	5.5 ± 8.4	1.7 ± 0.4
Summer	Males	6	0.02 ± 0.03	0.3 ± 0.4	0.05 ± 0.1	9.6 ± 12.1	2.4 ± 2.0	**0.03 ± 0.1**	1.2 ± 0.3	0.3 ± 0.3	4.4 ± 1.1	18.2 ± 14.0	7.2 ± 8.6	1.3 ± 0.3
Summer	Eggs	14	0.005 ± 0.01	**0.8 ± 0.4**	**0.4 ± 0.2**	34.0 ± 11.9	1.5 ± 1.2	0.0	**2.0 ± 0.6**	**1.6 ± 1.4**	**50.8 ± 19.7**	**91.2 ± 29.8**	16.9 ± 4.0	2.1 ± 0.5

^a^Area: Shrimp Fishing Area (SFAs).

^b^Number of total individuals per type used for lipid class analysis.

^c^TAG: Triacylglycerols.

^d^FFA: Free Fatty Acids.

^e^AMPL: Acetone Mobile Polar Lipids.

^f^PL: Phospholipids.

^g^TAG/ST: Triacylglycerols/ Sterols.

^h^EPA + DHA: 20:5ω-3 (EPA) + 22:6ω-3 (DHA)

**Fig 7:**
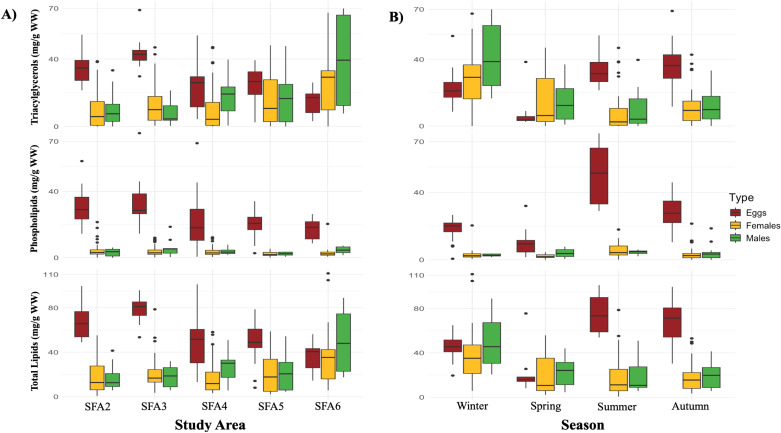
Seasonal and spatial variations in lipid classes and total lipid content in northern shrimp (*Pandalus borealis*). Triacylglycerol, phospholipid, and total lipid contents are measured in mg per g of wet weight (mg/g WW). The middle part of the box, or the “interquartile range,” represents the middle quartiles (or the 75th minus the 25th percentile). The black line in the box represents the median. The minimum and maximum values of the data are indicated by the upper and lower lines of the box, respectively. Points beyond the lines represent outliers in the data set.

Seasonal and regional variations in total lipid content and lipid classes were observed (Fig 7). TAG concentrations (given as mg/g WW) were significantly different among SFAs (p < 0.05) and seasons (p < 0.001), whereas PL content was markedly distinct among maturity stages (p < 0.001) and seasons (p < 0.001). Eggs consistently showed the highest PL levels year-round across all study areas. Additionally, TAG levels fluctuated with a notable increase in TAG amounts in male shrimp during winter and in SFA6. Shrimp eggs exhibited higher total lipid content, peaking in summer and the northernmost SFAs (Fig 7). From winter onwards, all shrimp tissues gradually decreased total lipid amounts, mirroring the decline observed in total lipid content in eggs from high to low latitudes. In this study, no relationship was found between the average weight and length of females and the total lipid content measured in their eggs. In addition to the lipid classes, higher EPA + DHA levels were observed in the eggs of ovigerous females across seasons, with peaks observed in autumn. Finally, high TAG/sterol ratio values were found in both females and males in the SFA6 and in the winter period (Table 3).

## 4. Discussion

*Pandalus borealis* exhibited distinct feeding patterns influenced by local and seasonal variability in environmental conditions and food availability. Trophic markers indicated that *P. borealis* fed on a combination of pelagic and benthic resources, with notable regional differences. During the autumn months, the shrimp expanded their ecological niche, with females displaying a broader spectrum of prey items. Changes in the feeding behavior were further evidenced by fluctuations in the content of lipid reserves. *Pandalus borealis* from northern regions exhibited higher levels of lipid reserves in eggs, which is likely to support egg survival overwinter, while shrimp from southern regions (SFA6) showed higher levels of lipid storage in muscle tissue, probably due to regional differences in food availability and energy allocation. Overall, *P. borealis* demonstrated remarkable dietary flexibility and opportunistic feeding strategies, allowing it to respond to changing environmental conditions and prey availability, thus reinforcing its important ecological role as a facilitator of energy transfer between trophic levels and habitats in sub-Arctic regions.

### 4.1. Feeding and foraging behaviour of northern shrimp

#### Relative contribution of carbon sources according to stable isotopes.

*Pandalus borealis* uses different trophic pathways in its diet in areas covered by this study according to the range of *δ*^13^C and *δ*^15^N values, which are a proxy for the total diversity of resources. On the one hand, *δ*^13^C values suggested that shrimp feed primarily on a mixture of pelagic and benthic sources, where according to Stein and Macdonald [63], *δ*^13^C values from these combined sources typically range from –24.0 ‰ to –19.0 ‰. The *δ*^13^C composition of shrimp revealed that they preyed on a wide range of isotopically different resources, suggesting changes in the composition of prey items used by shrimp among different SFAs. For example, the most ¹³C-enriched average isotopic composition in females of shrimp in SFA3 suggested a greater reliance on ice-derived carbon and/or the consumption of alternative sources, including reworked organic material [64]. In comparison, the most ¹³C-depleted values (–21.2 ‰) recorded in male shrimp in SFA2 denoted a diet based on phytoplankton sources. On the other hand, *δ*^15^N isotopic values showed that female shrimp not only exploited a wide variety of resources but also accessed prey from different trophic levels, including higher trophic levels compared to their male counterparts (Figs 3 and S1). From the isotopic values, it appears that *P. borealis* individuals do not always share similar diets and exhibit varied preferences for different food items. Accordingly, previous studies found that *P. borealis* males actively feed on plankton (*i.e*., ice-associated algae and/or phytoplankton) more than females [65]. This supports the interpretation that lower *δ*^15^N values in males are associated with herbivorous feeding behaviours, while higher *δ*^15^N values in females indicate predominantly omnivorous and carnivorous behaviours or the consumption of sinking reworked organic material. Furthermore, fluctuations in the isotopic composition may reflect an average of varying proportions of prey items assimilated by individuals over time [66] influenced by aspects such as prey availability, life stage, shifts in feeding activity (*i.e.*, vertical migrations), and inter- and intraspecific competition.

#### Variation in trophic niche structure.

Bayesian estimations of the standard ellipse area (SEAc) revealed variations in the isotopic niche width of *P. borealis* populations across spatial and seasonal gradients. Specifically, results showed a visually notable decrease in niche width from northwest to southeast SFAs and an increase in trophic niche size during the autumn (Fig 4). Because niche dimensions can rapidly adjust to variations in both intraspecific and interspecific competition and changes in prey abundance, the observed increase in niche width suggests that shrimp populations feed on a broader range of prey items or use prey across a wider spectrum of trophic levels [66]. In comparison, a narrower niche width in southeast SFAs indicates a reliance on a more limited range of food items or shifts in dietary preferences over time [66]. Furthermore, niche dimensions may reflect fluctuations in the degree of trophic specialization among individual shrimp in response to changes in environmental productivity [67,68]. Isotopic niche analysis (δ^13^C *vs.* δ^15^N biplots) among regions also revealed distinct differences in niche overlap. Notably, SFA3 exhibited no overlap with other fishing areas, implying that *P. borealis* in SFA3 may rely on distinct food sources or use different foraging behaviours compared to shrimp in other regions (See Figs 3 and 4A). Likewise, results revealed a smaller niche overlap between females and males in SFA3 compared to other regions (S1 Fig), suggesting a lower level of competition between individuals. This also suggests that both biotic and abiotic conditions in SFA3 may differ from those along the Labrador coast. Variation in niche size and separation suggest that *P. borealis* exhibits dietary flexibility, not relying on specific types of prey. Instead, this species likely adapts to opportunistic feeding strategies, adjusting its diet based on prey availability across different regions and seasons. This adaptability may reflect an ecological advantage, allowing this species to exploit a wider range of food resources in response to fluctuating environmental conditions, competition dynamics, and prey availability. This hypothesis is supported by stomach content analyses that reveal dynamic shifts in prey preferences of *P. borealis* over time and migratory patterns [5,6]. These studies revealed that shrimp diet encompasses a wide range of prey, including annelids, small crustaceans, phytoplankton, zooplankton, and detritus during diurnal activity periods. Conversely, nocturnal foraging predominantly targets copepods and euphausiids as primary prey sources [5,6]. This highlights the important role of *P. borealis* in sub-Arctic food webs by increasing the number of energy flow pathways between pelagic and benthic habitats and low-intermediate and high trophic levels.

#### Role of environmental variability in stable isotope and fatty acid compositions.

In sub-Arctic ecosystems, life strategies of various species are closely related to seasonality in environmental conditions and primary production. In particular, temperature and sea ice conditions considerably influence the time of blooms (ice-associated *vs.* pelagic) and the timing of organic material fluxes at different depths [67,69,70]. For instance, the increase in diatom abundance is strongly associated with rising temperatures and reduced sea ice concentration in early spring, whereas autotrophic flagellates remain consistently present throughout the year, unaffected by seasonal variations [70]. Thus, in seasonal oceans, environmental conditions may significantly influence dynamics in the biochemical composition of consumers by affecting the timing, abundance, and taxonomic composition of primary producers [71].

Stable isotope analyses in subpolar regions have shown a relationship between variations in carbon and nitrogen signatures and fluctuations in environmental conditions. For example, variability in *δ*^13^C signatures of benthic consumers was linked to variations in the composition of prey sources driven by changes in sea ice conditions [67]. In contrast, variations in *δ*^15^N signatures appear to be associated with depth gradients and alterations in the biochemical characteristics of sinking particles caused by abiotic degradation processes, mostly at the first meters of the water column [72,73]. Furthermore, anthropogenic nitrogen inputs, including runoff or atmospheric deposition, may also lead to isotopic variability in nitrogen sources, thereby affecting baseline isotopic values in marine food webs [57,74].

Environmental variables, including depth, pH, temperature, light intensity, and salinity, are also recognized for influencing lipid metabolism, composition, and storage of individual FAs in different marine groups [75–78]. In this study, the marked variability in SI composition and FA profiles observed across SFAs and seasons suggested that environmental conditions may significantly influence dynamics in the biochemical composition of ecosystems, thereby affecting trophic markers and FA composition of shrimp. For example, significant differences in dietary markers across continuous SFAs indicated that *P. borealis* rely on distinct food sources, in which habitat conditions play an essential role in shaping their availability (see the next section of the discussion). Linear models revealed a significant interaction effect between environmental variables (*i.e*., temperature, bottom depth, and sea ice concentration) and changes in both SI and FA profiles in *P. borealis*. For example, water temperature and bottom depth were the most significant factors affecting SAFA values, while depth had the greatest influence on MUFA and PUFA levels. These findings are consistent with previous research suggesting that such variations are linked to modulations in lipidome composition as a response to physical environmental stress (*e.g*., homeoviscous adaptation) [79,80]. In contrast, fluctuations in sea ice concentration and temperature were key factors affecting the total lipid content and lipid classes in shrimp. Recognizing the significant effect of environmental variables on lipid metabolism, further studies are needed to precisely determine the effects of a changing sub-Arctic ecosystem on the storage and transfer processes of essential lipids within high-latitude marine food webs. However, accurately determining the impact of specific environmental variables on lipid metabolism and storage is challenging due to the difficulty in isolating specific environmental drivers in this type of analysis [75].

#### Fatty acid composition among spatial and seasonal gradients.

In the present study, the highest proportions of the diatom-associated FAs (16:1ω7 and 20:5ω3; EPA), along with the zooplankton-associated FAs (18:1ω7, 18:1ω9, 20:1ω9, and 22:1ω11), and dinoflagellate-associated FA (22:6ω3; DHA), indicated a significant dietary contribution from both diatoms and zooplankton to shrimp. This FA composition aligns with profiles previously described by Ackman and Eaton [81] and Hopkins et al. [82] in which the SAFA 16:0, the MUFAs 16:1ω7 and 18:1ω9, and the PUFAs 20:5ω3 and 22:6ω3 dominated the lipid profiles in *P. borealis*. Shrimp eggs from Antarctic regions displayed lipid profiles closely aligning with our results, especially in the pronounced presence of 16:0, 16:1ω7, 18:1ω7, 18:1ω9 and 20:5ω3 as the dominant FAs [83]. The consistent dominance of these FAs over time and regions highlights the importance of these sources in shrimp diets, suggesting that *P. borealis* relies substantially on pelagic primary producers across different seasons and highlights the adaptability of this species to foraging strategies with diets based mainly on sinking phytodetritus. Interestingly, SI and FA analyses revealed a notable difference in the diet of *P. borealis* in SFA3 compared to those in other SFAs, including the nearby SFA2 (See Figs 4A and 5). In the present study, both biochemical trophic markers indicated that *P. borealis* in SFA3 were feeding primarily on a profile associated with benthic and land-sea margin habitats. This assumption was supported by their FA profiles, which showed higher proportions of 18:2ω6, typically associated with vascular plant markers [52], as well as significant amounts of 16:1ω7 and 18:1ω7 FAs, which can originate from either diatoms or bacteria [84]. In contrast, the presence of intact wax esters, as well as the high abundance of phytoplankton and *Calanus*-type markers 20:1 and 22:1, suggest a diet based on herbivorous copepods in SFA2 and SFA6 [47,85]. This study revealed that the FA composition of shrimp closely matches the lipid profiles of their prey, particularly zooplankton. This suggests that herbivorous diets, primarily based on phytoplankton species, play a crucial role in the rapid and efficient transfer and storage of high-energy lipids (*e.g*., triacylglycerols and wax esters) within sub-Arctic marine food webs. Since *P. borealis* seems to rely on diatoms and zooplankton as its main sources of lipids, our study highlights the vulnerability of this species to possible changes in seasonal production cycles as a result of anthropogenic climate change.

Seasonal storage of essential lipid reserves may be a requirement for marine crustaceans living in extreme environments that ensure short seasonal periods of food abundance [86]. *Pandalus borealis* tend to accumulate lipids during periods of high food availability, which are then utilized during spawning and larval development [82]. Thus, the timing of these productivity bloom cycles is fundamental for ensuring that larvae hatch with sufficient lipid reserves [53]. Since some marine invertebrates cannot produce certain FAs and must obtain them from their diet [21], variations in the lipid composition of *P. borealis* may reflect changes in their diets according to seasonal fluctuations in the composition of primary producers. In this study, FA analyses revealed high relative proportions of the diatom markers 16:1ω7, 20:4ω6, and 20:5ω3 in shrimp eggs during winter and spring. Notably, increases in levels of 20:4ω6 and 20:5ω3 in eggs were also accompanied by rises in PUFAs, omega-3, and DHA proportions in spring. Likewise, increases in the proportions of zooplankton markers and MUFAs were observed in both females and males during winter and spring. The increased contribution of diatom- and zooplankton-associated FAs in the shrimp diet underscores the reinforcement of the sympagic-pelagic-benthic coupling during these seasons and highlights the essential ecological role of these sources influencing diets and lipids dynamics in shrimp year-around. Lipid analyses revealed seasonal fluctuations in total lipid content within *P. borealis* tissues. Specifically, an increase in the amount of total lipids in shrimp eggs was observed between spring and summer, along with peaks in total lipid levels for both female and male shrimp during the winter season (Fig 7). These findings align with previous studies that reported similar seasonal patterns in total lipid content. Specifically, a greater accumulation of lipid reserves was observed during winter when food is scarce [82,87,88], as well as during reproductive processes, which are often linked to the spring phytoplankton bloom [88,89]. However, variations in total lipid content and the production of storage lipids may also be influenced by other drivers, including overwintering strategies, seasonal variability in feeding behaviour, and among maturity stages [87].

### 4.2. Nutritional status of northern shrimp

In high-latitude ecosystems, seasonal variability in environmental conditions such as temperature, light availability, and nutrient dynamics significantly impact the magnitude and duration of primary producer blooms [90]. These environmental shifts also play crucial roles in influencing the abundance and types of lipids synthesized by phytoplankton and other primary producers [71,91]. Consequently, it is predicted that global climate change could affect the lipid profiles of primary producers, impacting the production of EFAs such as omega-3 PUFAs, particularly EPA and DHA [92,93]. Reductions in the planktonic synthesis of omega-3 FAs will cascade through the food web, impacting the lipid intake and storage of essential lipids at higher trophic levels, which ultimately may affect the growth, reproduction, and survival of diverse species, including *P. borealis* [27].

The allocation of energy towards lipid storage is vital for many marine invertebrates, especially in cold and nutrient-variable environments [55]. Lipid reserves, especially TAGs, are important for energy-intensive processes like reproduction and larval development in crustaceans, making TAG content a key indicator of nutritional conditions [55,94]. For example, studies have shown that higher lipid reserves in female shrimp correlate with increased fecundity and better egg quality, which enhance larval survival [52]. Additionally, investigations concluded that during starvation or moulting phases the rapid catabolism of TAG results in changes in lipid class composition, reflected in declining TAG and TAG-sterol ratios. In contrast, TAG-sterol ratios typically increase throughout development periods due to the rapid accumulation of TAGs derived from external food intake [55]. Changes in TAG content (as mg/g WW) and TAG-sterol ratios were registered in spatiotemporal gradients in this study. On the one hand, our data exposed a gradual reduction in TAG and TAG-sterol ratios in the muscles of females and males from winter to summer, contrasting with increases in TAG and TAG-sterol ratios in eggs from spring to autumn. On the other hand, TAG and TAG-sterol ratios in eggs were higher in the northernmost areas (SFA2 and SFA3), whereas increases in TAG and TAG-sterol ratios in the muscles of males and females were recorded in the southernmost fishing area (SFA6). As food availability fluctuates spatially and temporally in sub-Arctic regions, lipid profiles, storage, and nutritional conditions of *P. borealis* are likely to adjust accordingly. Hence, variations in TAG content may result from fluctuations in the timing of pelagic production, reflecting periods of starvation or increased food intake [82,94]. In particular, in zooplankton-feeding species like *P. borealis*, fluctuations in TAG levels may be influenced by the availability of wax esters, which are biosynthesized by zooplankton and stored as energy reserves by consumers [95].

Lipid profiles in crustaceans may vary based on feeding conditions and between maturity stages, suggesting that male and female shrimp may allocate or deplete lipid reserves differently depending on physiological needs [94,96]. This study highlighted that eggs are significantly richer in essential lipids, including phospholipids and omega-3 FAs, compared to the lipid content in ovigerous females (see Table 3). It suggests that the production of lipid-rich eggs may impose considerable demands on the lipid reserves of females, a trend observed in other crustacean species as well [88]. For example, it was observed that wax esters are rapidly consumed during the development of fertilized eggs [95]. The energetic investment in egg production (measured as poor TAG content) could explain, in part, the relatively lipid-deficient and notable variability in lipid profiles observed in ovigerous females and their eggs. This study also revealed an increase in the storage of TAG, PL, and total lipids in eggs from summer to fall, suggesting that ovigerous females may allocate lipid reserves differently among tissues before winter. This dynamic adjustment in lipid storage may not only influence individual health and reproductive success in females of *P. borealis* but may also play an important role in population dynamics by influencing lipid conditions across various maturity stages, especially in larval individuals [82]. As larval individuals are particularly vulnerable to changes in food availability and environmental conditions, fluctuations in lipid storage can have cascading effects on survival rates and overall population stability [55]. Thus, understanding how interacting biogeochemical conditions influence lipid content and storage is crucial for predicting the responses of *P. borealis* populations to ongoing environmental changes in sub-Arctic ecosystems and for formulating effective conservation and management strategies.

### 4.3. Implications of climate change for shrimp stocks

As climate change intensifies, it increasingly disrupts trophic interactions and the foraging ecology of marine species in high-latitude regions by altering the abundance, distribution, and diversity of prey species, making the effects on ecosystem functioning more unpredictable [97]. The finding that the lipid content of eggs of *P. borealis* peaks prior to overwintering emphasizes the importance of synchronization between primary production blooms and the reproductive cycles of shrimp. Consequently, shifts in the onset of primary production could disrupt this synchrony, leading to a depletion of essential energy-rich lipid reserves that, in turn, could diminish reproductive output and larval survival, ultimately affecting population stocks of *P. borealis* [98]. However, regional differences in the ecological niche structure suggest that shrimp populations in various areas may respond differently to climate-induced changes in food availability, highlighting the remarkable dietary flexibility of this species in adapting to fluctuations in resource dynamics. In terms of fisheries management, this study highlights the importance of monitoring lipid-based condition indices, particularly during early life stages, as indicators of population health and recruitment potential [99]. Incorporating dietary tracer data into stock assessments can yield more accurate predictions of shrimp productivity under future climate scenarios. Finally, changes in the nutritional quality of *P. borealis* as a food source may alter its role as a key forage species for higher trophic levels, potentially impacting the net transfer of essential lipids in sub-Arctic ecosystems.

## Supporting information

S1 Fig Stable isotope biplots illustrating changes in the isotopic niche structure of the northern shrimp (*Pandalus borealis*) population in Canada’s sub-Arctic. The positions occupied by females and males of shrimp in the isotopic space are represented by dots in each δ^13^C - δ^15^N biplot. Standard ellipses (solid lines) enclose the size-corrected standard ellipse area (SEAc, fits 40% of the data) of shrimp along the shrimp fishing areas (SFAs).
(TIFF)

S2 Fig Spatial and temporal variation in fatty acid composition of zooplankton and northern shrimp (*Pandalus borealis*) in Canada’s sub-Arctic regions. Fatty acid profiles of northern shrimp collected from various management regions, including the Labrador Shelf (LS) and shrimp fishing areas (SFAs 2–6), highlight spatial variation (A). Fatty acid profiles grouped by season illustrate temporal differences in FA composition (B). The middle part of the box, or the “interquartile range,” represents the middle quartiles (or the 75th minus the 25th percentile). The black line in the box represents the median. The minimum and maximum values of the data are indicated by the upper and lower lines of the box, respectively. Points beyond the lines represent outliers in the data set.
(TIFF)

S1 Table Sampling details of the sites where northern shrimp (*Pandalus borealis*) was collected between 2022 and 2023 across five fishing areas in Canada’s sub-Arctic. (DOCX)

S2 Table Isotopic composition of northern shrimp (*Pandalus borealis*) in Canada’s sub-Arctic regions. (DOCX)

S3 Table Summary of the main effects on stable isotope (δ^13^C-δ^15^N) values of northern shrimp (*Pandalus borealis*) across five shrimp fishing areas in Canada’s sub-Arctic regions. (DOCX)

S4 Table Summary of the main effects on saturated, monounsaturated and polyunsaturated fatty acid values of northern shrimp (*Pandalus borealis*) across five shrimp fishing areas in Canada’s sub-Arctic regions. (DOCX)

S5 Table Summary of the main effects on lipid classes and total lipid content values of northern shrimp (*Pandalus borealis*) across five shrimp fishing areas in Canada’s sub-Arctic regions. (DOCX)

S6 Table Relative abundance of individual fatty acids as a percentage of total FAs (mean values with standard deviations) in zooplankton in Canada’s sub-Arctic regions. Zooplankton samples were collected south to north from various stations including Sentinel, Isecold-1, Isecold-2, SagBank, Hatton Basin, Isecold-3, Killinek Main, and Hatton 600. EPA: polyunsaturated eicosapentaenoic acid; DHA: polyunsaturated docosahexaenoic acid; SFA: saturated FA; MUFA: monounsaturated FA; PUFA: polyunsaturated FA; ω3: Omega-3; ω6: Omega-6, and EFA: essential fatty acids (20:4ω-6; 20:5ω-3; 22:6ω-3). Only those fatty acids ≥1% are included in this table.
(DOCX)

S7 Table The relative concentration of lipid classes in zooplankton in Canada’s sub-Arctic regions. The average lipid class contents (as mg/g WW) are measured in milligrams per gram of wet weight.
(DOCX)

S8 Table Isotopic composition of zooplankton across Canada’s sub-Arctic regions. (DOCX)

## Abbreviations

FAs: fatty acids
EFAs: essential fatty acids
SAFAs: saturated fatty acids
MUFAs: monounsaturated fatty acids
PUFAs: polyunsaturated fatty acids
FFA: free fatty acids
DHA: docosahexaenoic acid
EPA: eicosapentaenoic acid
ST: sterols
PL: phospholipids
TAG: triacylglycerols
SI: stable isotopes
SFA: shrimp fishing area
SIC: sea ice concentration.
