# Supplementary figures and images for: Trophic ecology and nutritional status of northern shrimp in Canada’s sub-Arctic
Source: PLoS One. 2025 May 20;20(5):e0322745. doi: 10.1371/journal.pone.0322745 (PMC12091755; doi:10.1371/journal.pone.0322745)

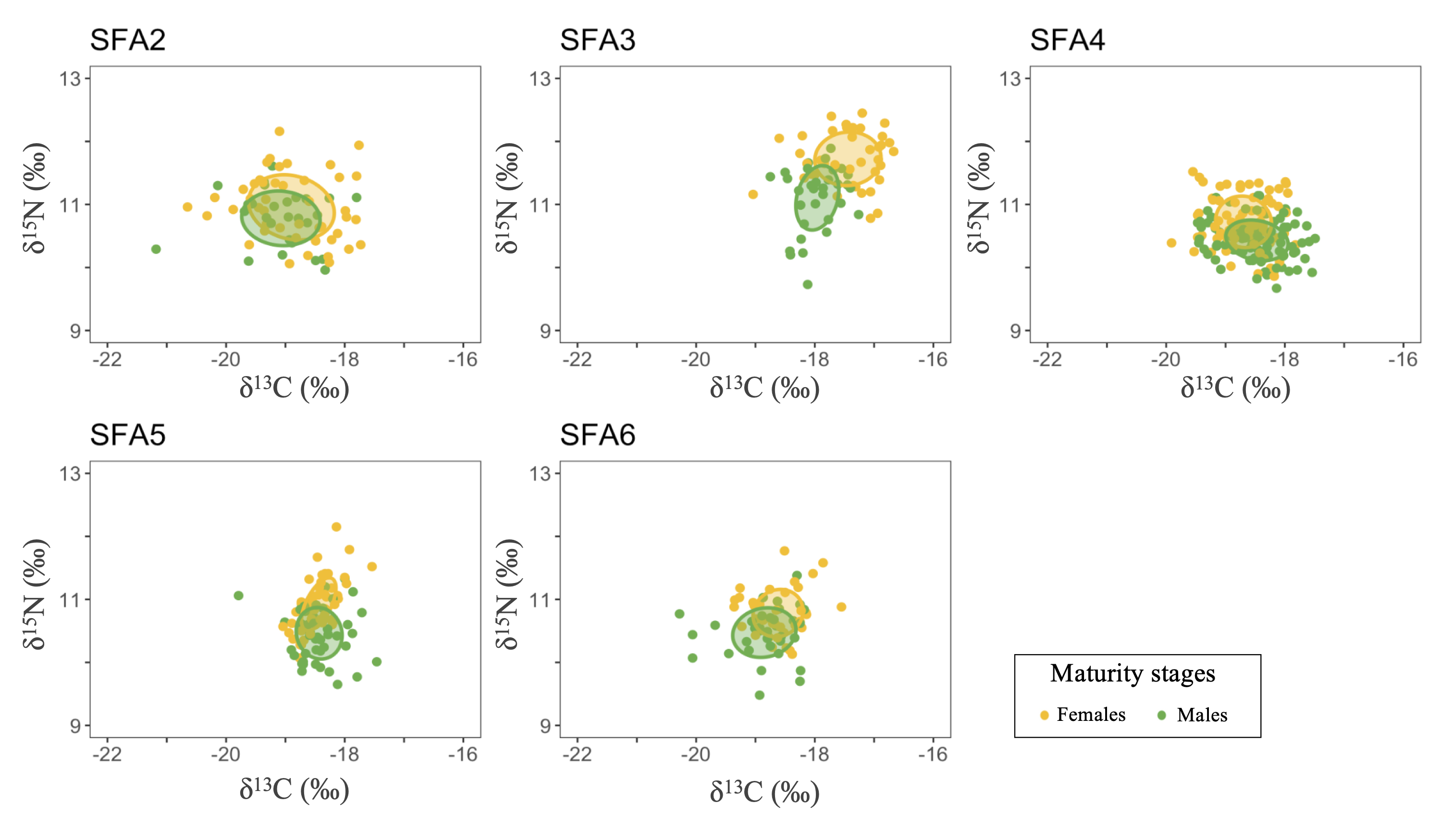

Supplement: S1 Fig — The positions occupied by females and males of shrimp in the isotopic space are represented by dots in each δ13C - δ15N biplot. Standard ellipses (solid lines) enclose the size-corrected standard ellipse area (SEAc, fits 40% of the data) of shrimp along the shrimp fishing areas (SFAs). (TIFF) [file pone.0322745.s001.tiff]

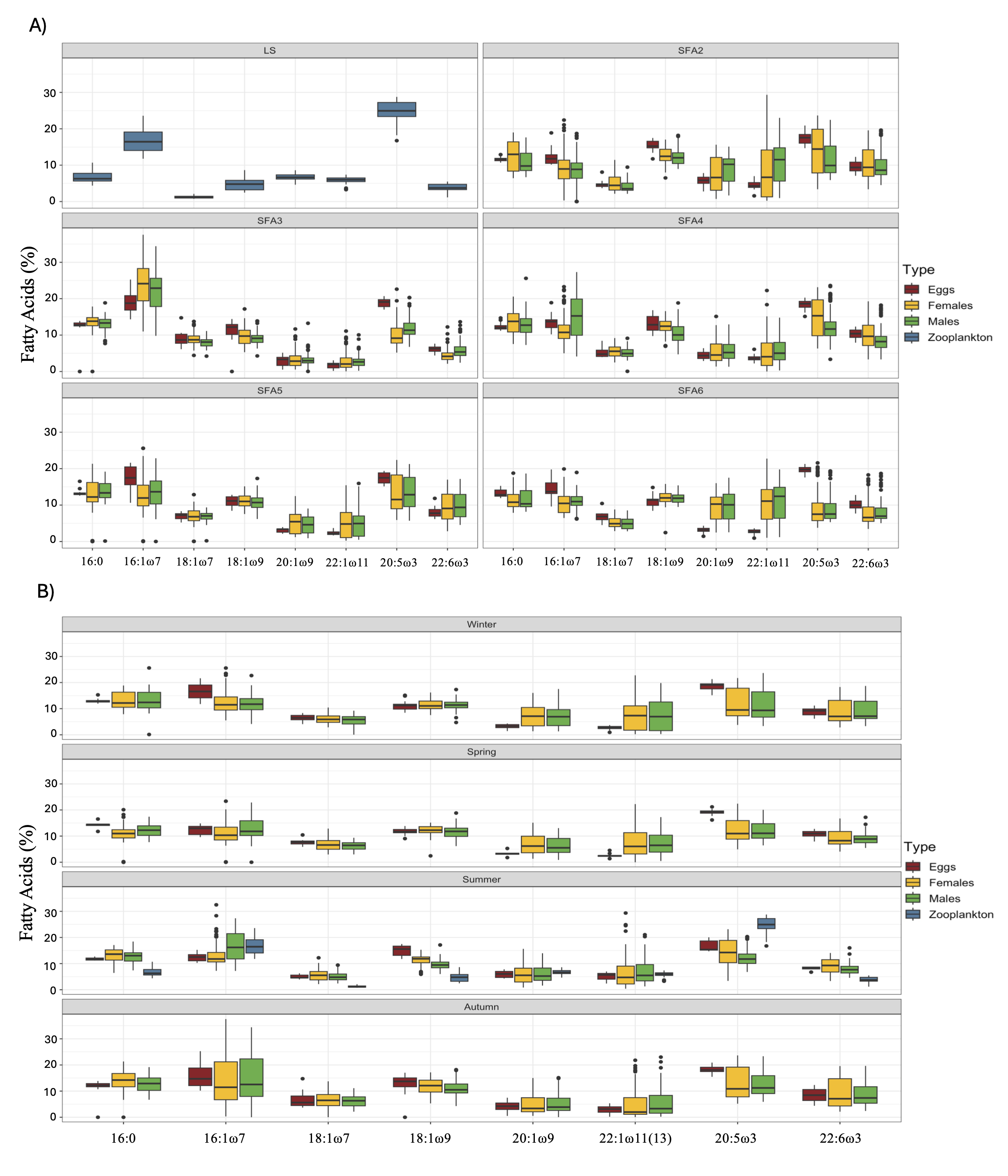

Supplement: S2 Fig — Fatty acid profiles of northern shrimp collected from various management regions, including the Labrador Shelf (LS) and shrimp fishing areas (SFAs 2–6), highlight spatial variation (A). Fatty acid profiles grouped by season illustrate temporal differences in FA composition (B). The middle part of the box, or the “interquartile range,” represents the middle quartiles (or the 75th minus the 25th percentile). The black line in the box represents the median. The minimum and maximum values of the data are indicated by the upper and lower lines of the box, respectively. Points beyond the lines represent outliers in the data set. (TIFF) [file pone.0322745.s002.tiff]
