# Supplementary material for: Trophic ecology and nutritional status of northern shrimp in Canada’s sub-Arctic
Source: PLoS One. 2025 May 20;20(5):e0322745. doi: 10.1371/journal.pone.0322745 (PMC12091755; doi:10.1371/journal.pone.0322745)
Supplement: S1 Table — (DOCX) [file pone.0322745.s003.docx]

**S1 Table.** Sampling details of the sites where northern shrimp (*Pandalus borealis*) was collected between 2022 and 2023 across five fishing areas in Canada’s sub-Arctic.

| **Shrimp**  **Fishing Area** | **Date Sampling^a^** | **Season** | **Type** | **n**^b^ | **Maturity**  **Code**^c^ | **Total Weight (wet/frozen)**  (mg) | **Carapace Length**  (mm) | **Total Length** (mm) | **Latitude**  (N) | **Longitude**  (W) | **Depth**  (m) | **Bottom Temp.**  (°C) | **Surface**  **Temp.**  (°C) | **SIC^d^** |
| --- | --- | --- | --- | --- | --- | --- | --- | --- | --- | --- | --- | --- | --- | --- |
| SFA2 | 2022-10-23 | Autumn | Ovigerous | 11 | 9811 | 12.2 ± 1.4 | 27.3 ± 0.7 | 119.7 ± 8.0 | 61.10 | –64.12 | 333 | 3.5 | 0.9 | 1.5 |
| SFA2 | 2022-10-23 | Autumn | Males | 9 | 9801 | 6.6 ± 1.3 | 22.7 ± 1.5 | 94.5 ± 9.6 | 61.10 | –64.12 | 333 | 3.5 | 0.9 | 1.5 |
| SFA2 | 2022-11-22 | Autumn | Ovigerous | 3 | 9811 | 12.8 ± 1.7 | 26.2 ± 2.1 | 124.7 ± 9.3 | 61.21 | –64.21 | 372 | 3.6 | –0.7 | 0.0 |
| SFA2 | 2022-11-22 | Autumn | Males | 5 | 9801 | 6.8 ± 1.6 | 21.8 ± 3.6 | 104.7 ± 17.6 | 61.21 | –64.21 | 372 | 3.6 | –0.7 | 0.0 |
| SFA2 | 2022-12-06 | Autumn | Ovigerous | 11 | 9811 | 12.7 ± 1.4 | 26.8 ± 0.7 | 112.0 ± 8.6 | 60.75 | –64.10 | 390 | 4.0 | –1.6 | 0.0 |
| SFA2 | 2022-12-06 | Autumn | Males | 9 | 9801 | 7.2 ± 1.4 | 23.5 ± 1.9 | 97.7 ± 11.1 | 60.75 | –64.10 | 390 | 4.0 | –1.6 | 0.0 |
| SFA2 | 2022-12-08 | Autumn | Ovigerous | 11 | 9811 | 11.9 ± 1.0 | 26.8 ± 1.0 | 101.9 ± 5.8 | 60.88 | –63.99 | 400 | 4.3 | –1.6 | 1.6 |
| SFA2 | 2022-12-08 | Autumn | Males | 9 | 9801 | 6.0 ± 0.9 | 22.9 ± 1.1 | 84.7 ± 4.9 | 60.88 | –63.99 | 400 | 4.3 | –1.6 | 1.6 |
| SFA2 | 2022-12-08 | Autumn | Ovigerous | 11 | 9811 | 11.9 ± 1.4 | 26.6 ± 1.5 | 113.8 ± 4.3 | 60.80 | –63.63 | 465 | 4.7 | –1.6 | 0.8 |
| SFA2 | 2022-12-08 | Autumn | Males | 9 | 9801 | 8.1 ± 1.3 | 23.7 ± 1.8 | 99.6 ± 5.9 | 60.80 | –63.63 | 465 | 4.7 | –1.6 | 0.8 |
| SFA2 | 2022-12-12 | Autumn | Ovigerous | 2 | 9811 | 10.2 ± 1.8 | 25.4 ± 0.6 | 106.7 ± 1.3 | 60.64 | –64.10 | 366 | 4.0 | –1.5 | 0.0 |
| SFA2 | 2022-12-12 | Autumn | Females | 7 | 9810 | 8.6 ± 1.1 | 24.4 ± 1.3 | 92.1 ± 5.6 | 60.64 | –64.10 | 366 | 4.0 | –1.5 | 0.0 |
| SFA2 | 2022-12-12 | Autumn | Males | 9 | 9801 | 4.6 ± 1.0 | 19.9 ± 1.7 | 81.8 ± 9.1 | 60.64 | –64.10 | 366 | 4.0 | –1.5 | 0.0 |
| SFA2 | 2023-07-09 | Summer | Females | 15 | 9806 | 8.7 ± 0.8 | 24.5 ± 1.2 | 101.4 ± 3.1 | 61.18 | –64.28 | 364 | 2.8 | 3.3 | 89.8 |
| SFA2 | 2023-08-22 | Summer | Ovigerous | 5 | 9811 | 10.0 ± 1.9 | 26.4 ± 2.1 | 114.8 ± 12.0 | 61.63 | –64.10 | 266 | 4.6 | 5.2 | 52.2 |
| SFA2 | 2023-08-22 | Summer | Males | 7 | 9801 | 3.6 ± 0.2 | 18.2 ± 0.7 | 90.7 ± 5.8 | 61.63 | –64.10 | 266 | 4.6 | 5.2 | 52.2 |
| SFA2 | 2023-08-30 | Summer | Ovigerous | 3 | 9811 | 10.6 ± 1.1 | 26.9 ± 1.2 | 118.2 ± 3.0 | 61.25 | –64.25 | 376 | 3.5 | 4.4 | 55.8 |
| SFA2 | 2023-08-30 | Summer | Females | 7 | 9809 | 11.1 ± 1.1 | 27.6 ± 1.3 | 113.3 ± 8.3 | 61.25 | –64.25 | 376 | 3.5 | 4.4 | 55.8 |
| SFA2 | 2023-08-30 | Summer | Males | 11 | 9801 | 5.0 ± 0.8 | 20.9 ± 0.9 | 95.4 ± 7.1 | 61.25 | –64.25 | 376 | 3.5 | 4.4 | 55.8 |
| SFA2 | 2023-09-06 | Summer | Ovigerous | 3 | 9811 | 11.2 ± 1.7 | 25.8 ± 1.7 | 120.1 ± 10.7 | 61.53 | –64.10 | 301 | 4.6 | 2.9 | 21.2 |
| SFA2 | 2023-09-06 | Summer | Females | 2 | 9809 | 9.9 ± 1.3 | 24.6 ± 0.4 | 112.7 ± 2.1 | 61.53 | –64.10 | 301 | 4.6 | 2.9 | 21.2 |
| SFA2 | 2023-09-06 | Summer | Males | 6 | 9801 | 3.9 ± 0.2 | 18.8 ± 0.9 | 92.0 ± 2.3 | 61.53 | –64.10 | 301 | 4.6 | 2.9 | 21.2 |
| SFA3 | 2022-10-10 | Autumn | Ovigerous | 13 | 9811 | 9.2 ± 1.7 | 23.9 ± 1.7 | 104.5 ± 8.6 | 60.37 | –65.94 | 380 | –0.4 | 2.5 | 0.0 |
| SFA3 | 2022-10-10 | Autumn | Males | 9 | 9801 | 4.0 ± 0.9 | 18.2 ± 1.8 | 83.2 ± 7.6 | 60.37 | –65.94 | 380 | –0.4 | 2.5 | 0.0 |
| SFA3 | 2022-11-10 | Autumn | Ovigerous | 13 | 9811 | 8.2 ± 1.4 | 22.3 ± 1.3 | 101.3 ± 5.6 | 60.37 | –65.82 | 368 | –0.3 | 0.5 | 0.0 |
| SFA3 | 2022-11-10 | Autumn | Males | 9 | 9801 | 4.7 ± 0.5 | 19.5 ± 1.0 | 85.0 ± 5.6 | 60.37 | –65.82 | 368 | –0.3 | 0.5 | 0.0 |
| SFA3 | 2022-11-12 | Autumn | Ovigerous | 12 | 9811 | 8.2 ± 0.7 | 22.3 ± 1.1 | 91.7 ± 5.8 | 61.09 | –68.87 | 371 | 2.6 | –0.6 | 0.0 |
| SFA3 | 2022-11-12 | Autumn | Males | 9 | 9801 | 4.2 ± 0.4 | 18.1 ± 1.0 | 85.5 ± 5.1 | 61.09 | –68.87 | 371 | 2.6 | –0.6 | 0.0 |
| SFA3 | 2022-12-11 | Autumn | Ovigerous | 9 | 9811 | 7.8 ± 1.0 | 22.4 ± 1.6 | 103.6 ± 11.2 | 60.49 | –65.85 | 364 | 0.0 | –1.3 | 0.0 |
| SFA3 | 2022-12-11 | Autumn | Females | 5 | 9810 | 7.9 ± 1.0 | 24.0 ± 0.7 | 102.6 ± 1.5 | 60.49 | –65.85 | 364 | 0.0 | –1.3 | 0.0 |
| SFA3 | 2022-12-11 | Autumn | Males | 20 | 9801 | 3.4 ± 0.6 | 17.2 ± 1.4 | 75.7 ± 7.7 | 60.49 | –65.85 | 364 | 0.0 | –1.3 | 0.0 |
| SFA3 | 2023-09-01 | Summer | Ovigerous | 9 | 9811 | 10.4 ± 1.5 | 24.4 ± 1.3 | 103.3 ± 6.0 | 60.27 | –65.94 | 319 | –0.8 | 4.4 | 19.8 |
| SFA3 | 2023-09-01 | Summer | Males | 12 | 9801 | 3.7 ± 0.5 | 18.2 ± 1.2 | 80.1 ± 3.8 | 60.27 | –65.94 | 319 | –0.8 | 4.4 | 19.8 |
| SFA3 | 2023-10-06 | Autumn | Ovigerous | 7 | 9811 | 9.4 ± 1.9 | 24.4 ± 2.1 | 108.7 ± 9.4 | 60.34 | –65.87 | 365 | –0.2 | 3.2 | 0.8 |
| SFA3 | 2023-10-06 | Autumn | Females | 2 | 9810 | 7.4 ± 0.0 | 22.9 ± 0.3 | 101.0 ± 4.7 | 60.34 | –65.87 | 365 | –0.2 | 3.2 | 0.8 |
| SFA3 | 2023-10-06 | Autumn | Males | 10 | 9801 | 4.3 ± 0.6 | 19.4 ± 1.3 | 83.9 ± 3.7 | 60.34 | –65.87 | 365 | –0.2 | 3.2 | 0.8 |

**^a^** Sampling date (year/month/day).

**^b^** Number of total individuals per station and maturity stage used for trophic marker analyses.

**^c^** Sexual maturity stages codes: **9801** - Male; **9806** - Female (1^st^-time spawner) with head roe; **9809** - Female (multiple spawner) with head roe; **9810** - Female (multiple spawner) no head roe; **9811** - Ovigerous Female.

^d^SIC: Average of sea ice concentration three months before sampling

**S1 Table.** Sampling details of the sites where northern shrimp (*Pandalus borealis*) was collected between 2022 and 2023 across five fishing areas in Canada’s sub-Arctic. This table is a continuation of Table 1.

| **Shrimp Fishing Area** | **Date Sampling^a^** | **Season** | **Type** | **n**^b^ | **Maturity**  **Code**^c^ | **Total Weight (wet/frozen)**  (mg) | **Carapace Length**  (mm) | **Total Length** (mm) | **Latitude**  (N) | **Longitude**  (W) | **Depth**  (m) | **Bottom Temp.**  (°C) | **Surface**  **Temp.**  (°C) | **SIC^d^** |
| --- | --- | --- | --- | --- | --- | --- | --- | --- | --- | --- | --- | --- | --- | --- |
| SFA4 | 2022-11-11 | Autumn | Ovigerous | 12 | 9811 | 11.4 ± 1.2 | 28.1 ± 1.0 | 109.0 ± 10.0 | 60.16 | –61.61 | 253 | 3.5 | 0.3 | 0.0 |
| SFA4 | 2022-11-11 | Autumn | Males | 10 | 9801 | 7.3 ± 1.0 | 24.6 ± 1.5 | 97.7 ± 10.7 | 60.16 | –61.61 | 253 | 3.5 | 0.3 | 0.0 |
| SFA4 | 2022-11-27 | Autumn | Ovigerous | 4 | 9811 | 10.8 ± 0.6 | 24.8 ± 1.0 | 114.4 ± 5.3 | 60.25 | –61.97 | 268 | 3.3 | 0.4 | 0.0 |
| SFA4 | 2022-11-27 | Autumn | Males | 8 | 9801 | 3.8 ± 0.4 | 17.5 ± 1.1 | 83.9 ± 10.5 | 60.25 | –61.97 | 268 | 3.3 | 0.4 | 0.0 |
| SFA4 | 2022-12-13 | Autumn | Ovigerous | 11 | 9811 | 10.0 ± 1.5 | 25.2 ± 1.4 | 99.4 ± 7.8 | 58.33 | –59.99 | 243 | 3.7 | –0.5 | 0.0 |
| SFA4 | 2022-12-13 | Autumn | Males | 9 | 9801 | 5.2 ± 0.8 | 21.2 ± 1.6 | 88.2 ± 8.3 | 58.33 | –59.99 | 243 | 3.7 | –0.5 | 0.0 |
| SFA4 | 2023-01-15 | Winter | Ovigerous | 11 | 9811 | 9.5 ± 1.9 | 25.3 ± 2.1 | 102.9 ± 9.3 | 58.30 | –59.80 | 387 | 4.4 | –0.7 | 0.0 |
| SFA4 | 2023-01-15 | Winter | Males | 8 | 9801 | 4.8 ± 0.6 | 21.0 ± 1.2 | 86.2 ± 3.9 | 58.30 | –59.80 | 387 | 4.4 | –0.7 | 0.0 |
| SFA4 | 2023-03-24 | Spring | Ovigerous | 9 | 9811 | 11.2 ± 1.7 | 26.4 ± 1.6 | 112.8 ± 11.9 | 58.23 | –59.83 | 287 | 4.0 | –1.7 | 32.1 |
| SFA4 | 2023-03-24 | Spring | Males | 9 | 9801 | 7.5 ± 0.8 | 24.1 ± 0.9 | 96.7 ± 8.6 | 58.23 | –59.83 | 287 | 4.0 | –1.7 | 32.1 |
| SFA4 | 2023-04-27 | Spring | Ovigerous | 11 | 9811 | 10.3 ± 1.0 | 25.8 ± 0.8 | 105.3 ± 6.3 | 58.30 | –60.03 | 229 | 2.6 | –1.6 | 54.1 |
| SFA4 | 2023-04-27 | Spring | Males | 9 | 9801 | 5.5 ± 0.6 | 20.6 ± 1.1 | 100.7 ± 2.7 | 58.30 | –60.03 | 229 | 2.6 | –1.6 | 54.1 |
| SFA4 | 2023-06-28 | Summer | Ovigerous | 13 | 9811 | 8.8 ± 1.3 | 24.4 ± 1.5 | 94.1 ± 4.9 | 58.47 | –60.00 | 310 | 0.3 | 0.4 | 46.3 |
| SFA4 | 2023-06-28 | Summer | Males | 15 | 9801 | 4.7 ± 0.7 | 19.8 ± 1.0 | 84.9 ± 5.0 | 58.47 | –60.00 | 310 | 0.3 | 0.4 | 46.3 |
| SFA4 | 2023-07-03 | Summer | Females | 13 | 9806 | 9.5 ± 1.2 | 24.6 ± 1.6 | 102.5 ± 4.9 | 57.98 | –59.80 | 287 | 3.1 | 5.2 | 50.3 |
| SFA4 | 2023-07-03 | Summer | Males | 15 | 9801 | 5.2 ± 0.7 | 19.7 ± 1.0 | 82.7 ± 5.1 | 57.98 | –59.80 | 287 | 3.1 | 5.2 | 50.3 |
| SFA4 | 2023-07-15 | Summer | Females | 14 | 9806 | 7.5 ± 0.9 | 22.4 ± 1.5 | 95.2 ± 5.6 | 60.30 | –61.63 | 287 | 2.7 | 3.9 | 73.1 |
| SFA4 | 2023-07-15 | Summer | Males | 15 | 9801 | 4.9 ± 0.8 | 19.3 ± 1.6 | 86.6 ± 3.6 | 60.30 | –61.63 | 287 | 2.7 | 3.9 | 73.1 |
| SFA4 | 2023-08-06 | Summer | Ovigerous | 13 | 9811 | 8.5 ± 1.4 | 24.1 ± 1.0 | 100.6 ± 7.7 | 58.20 | –59.88 | 238 | 2.2 | 6.0 | 38.6 |
| SFA4 | 2023-08-06 | Summer | Males | 14 | 9801 | 3.6 ± 0.3 | 18.2 ± 0.9 | 87.8 ± 7.8 | 58.20 | –59.88 | 238 | 2.2 | 6.0 | 38.6 |
| SFA4 | 2023-09-09 | Summer | Ovigerous | 7 | 9811 | 9.1 ± 0.7 | 25.1 ± 0.4 | 104.0 ± 8.4 | 60.23 | –61.73 | 257 | 3.7 | 2.8 | 30.7 |
| SFA4 | 2023-09-09 | Summer | Females | 3 | 9809 | 9.6 ± 1.0 | 26.0 ± 1.7 | 107.5 ± 2.4 | 60.23 | –61.73 | 257 | 3.7 | 2.8 | 30.7 |
| SFA4 | 2023-09-09 | Summer | Males | 11 | 9801 | 5.7 ± 1.2 | 21.9 ± 1.7 | 91.8 ± 6.1 | 60.23 | –61.73 | 257 | 3.7 | 2.8 | 30.7 |
| SFA5 | 2022-11-29 | Autumn | Ovigerous | 11 | 9811 | 9.7 ± 1.8 | 25.0 ± 2.0 | 106.6 ± 9.8 | 56.68 | –60.14 | 307 | 3.5 | 1.0 | 0.0 |
| SFA5 | 2022-11-29 | Autumn | Males | 9 | 9801 | 5.7 ± 1.7 | 21.0 ± 2.0 | 95.4 ± 11.6 | 56.68 | –60.14 | 307 | 3.5 | 1.0 | 0.0 |
| SFA5 | 2022-12-27 | Winter | Ovigerous | 11 | 9811 | 9.9 ± 1.5 | 24.3 ± 1.3 | 108.3 ± 9.5 | 56.48 | –59.75 | 252 | 0.1 | –0.1 | 0.0 |
| SFA5 | 2022-12-27 | Winter | Males | 9 | 9801 | 6.7 ± 0.8 | 21.4 ± 1.1 | 100.8 ± 6.0 | 56.48 | –59.75 | 252 | 0.1 | –0.1 | 0.0 |
| SFA5 | 2023-01-27 | Winter | Ovigerous | 9 | 9811 | 9.8 ± 2.4 | 24.3 ± 2.3 | 104.8 ± 15.2 | 56.47 | –59.77 | 354 | 0.4 | –1.5 | 0.0 |
| SFA5 | 2023-01-27 | Winter | Females | 2 | 9809 | 8.8 ± 1.8 | 25.5 ± 1.6 | 103.1 ± 1.3 | 56.47 | –59.77 | 354 | 0.4 | –1.5 | 0.0 |
| SFA5 | 2023-01-27 | Winter | Males | 9 | 9801 | 5.5 ± 0.9 | 20.8 ± 1.4 | 89.8 ± 10.2 | 56.47 | –59.77 | 354 | 0.4 | –1.5 | 0.0 |
| SFA5 | 2023-02-09 | Winter | Ovigerous | 11 | 9811 | 10.1 ± 1.6 | 24.7 ± 1.4 | 102.3 ± 12.7 | 54.92 | –55.98 | 317 | 3.8 | –1.5 | 1.6 |
| SFA5 | 2023-02-09 | Winter | Males | 9 | 9801 | 4.8 ± 1.0 | 20.0 ± 1.4 | 88.6 ± 4.9 | 54.92 | –55.98 | 317 | 3.8 | –1.5 | 1.6 |
| SFA5 | 2023-02-13 | Winter | Ovigerous | 9 | 9811 | 9.3 ± 2.0 | 23.1 ± 2.0 | 104.1 ± 7.7 | 54.85 | –56.01 | 372 | 3.8 | –1.6 | 1.5 |
| SFA5 | 2023-02-13 | Winter | Males | 9 | 9801 | 2.9 ± 0.7 | 16.5 ± 1.4 | 77.0 ± 5.4 | 54.85 | –56.01 | 372 | 3.8 | –1.6 | 1.5 |
| SFA5 | 2023-04-25 | Spring | Ovigerous | 10 | 9811 | 7.4 ± 1.3 | 22.8 ± 1.2 | 96.2 ± 4.1 | 55.03 | –56.03 | 327 | 3.3 | –1.5 | 41.5 |
| SFA5 | 2023-04-25 | Spring | Males | 10 | 9801 | 2.8 ± 0.6 | 16.3 ± 1.3 | 71.7 ± 5.9 | 55.03 | –56.03 | 327 | 3.3 | –1.5 | 41.5 |

**^a^** Sampling date (year/month/day).

**^b^** Number of total individuals per station and maturity stage used for trophic marker analyses.

**^c^** Sexual maturity stages codes: **9801** - Male; **9806** - Female (1^st^-time spawner) with head roe; **9809** - Female (multiple spawner) with head roe; **9810** - Female (multiple spawner) no head roe; **9811** - Ovigerous Female.

^d^SIC: Average of sea ice concentration three months before sampling

**S1 Table.** Sampling details of the sites where northern shrimp (*Pandalus borealis*) was collected between 2022 and 2023 across five fishing areas in Canada’s sub-Arctic. This table is a continuation of Table 1.

| **Shrimp Fishing Area** | **Date Sampling^a^** | **Season** | **Type** | **n**^b^ | **Maturity**  **Code**^c^ | **Total Weight (wet/frozen)**  (mg) | **Carapace Length**  (mm) | **Total Length** (mm) | **Latitude**  (N) | **Longitude**  (W) | **Depth**  (m) | **Bottom Temp.**  (°C) | **Surface**  **Temp.**  (°C) | **SIC^d^** |
| --- | --- | --- | --- | --- | --- | --- | --- | --- | --- | --- | --- | --- | --- | --- |
| SFA5 | 2023-04-27 | Spring | Ovigerous | 10 | 9811 | 9.0 ± 1.5 | 23.5 ± 1.2 | 101.6 ± 9.2 | 54.69 | –55.90 | 348 | 3.1 | –1.6 | 50.8 |
| SFA5 | 2023-04-27 | Spring | Males | 10 | 9801 | 4.2 ± 0.5 | 18.8 ± 0.8 | 84.3 ± 6.4 | 54.69 | –55.90 | 348 | 3.1 | –1.6 | 50.8 |
| SFA5 | 2023-05-07 | Spring | Ovigerous | 8 | 9811 | 9.6 ± 1.7 | 23.8 ± 1.6 | 102.8 ± 8.2 | 56.20 | –59.10 | 287 | 2.2 | –0.6 | 69.7 |
| SFA5 | 2023-05-07 | Spring | Males | 10 | 9801 | 3.5 ± 0.8 | 17.1 ± 1.2 | 80.8 ± 7.7 | 56.20 | –59.10 | 287 | 2.2 | –0.6 | 69.7 |
| SFA5 | 2023-06-12 | Spring | Females | 9 | 9806 | 9.0 ± 1.3 | 23.6 ± 1.6 | 99.7 ± 5.0 | 56.53 | –59.90 | 356 | 0.2 | 0.4 | 86.8 |
| SFA5 | 2023-06-12 | Spring | Males | 9 | 9801 | 3.1 ± 0.5 | 16.2 ± 0.9 | 76.0 ± 9.2 | 56.53 | –59.90 | 356 | 0.2 | 0.4 | 86.8 |
| SFA6 | 2023-01-16 | Winter | Ovigerous | 9 | 9811 | 9.9 ± 1.2 | 22.9 ± 1.3 | 102.6 ± 5.5 | 52.25 | –53.79 | 392 | 3.0 | –0.3 | 0.0 |
| SFA6 | 2023-01-16 | Winter | Males | 9 | 9801 | 5.2 ± 1.0 | 19.0 ± 1.4 | 88.2 ± 7.8 | 52.25 | –53.79 | 392 | 3.0 | –0.3 | 0.0 |
| SFA6 | 2023-01-30 | Winter | Ovigerous | 3 | 9811 | 10.3 ± 0.5 | 23.5 ± 1.4 | 125.3 ± 11.4 | 52.29 | –53.87 | 346 | 2.8 | –0.4 | 0.0 |
| SFA6 | 2023-01-30 | Winter | Males | 5 | 9801 | 4.8 ± 0.9 | 18.9 ± 1.4 | 91.7 ± 9.4 | 52.29 | –53.87 | 346 | 2.8 | –0.4 | 0.0 |
| SFA6 | 2023-01-31 | Winter | Ovigerous | 12 | 9811 | 10.8 ± 1.5 | 25.4 ± 1.3 | 114.9 ± 9.4 | 52.38 | –53.65 | 362 | 2.4 | –0.3 | 0.0 |
| SFA6 | 2023-01-31 | Winter | Males | 9 | 9801 | 4.4 ± 0.8 | 20.1 ± 1.3 | 82.1 ± 5.3 | 52.38 | –53.65 | 362 | 2.4 | –0.3 | 0.0 |
| SFA6 | 2023-02-11 | Winter | Ovigerous | 9 | 9811 | 9.1 ± 1.4 | 23.5 ± 1.5 | 97.5 ± 9.4 | 52.30 | –53.84 | 374 | 3.1 | –1.5 | 0.0 |
| SFA6 | 2023-02-11 | Winter | Males | 9 | 9801 | 3.3 ± 0.7 | 17.1 ± 1.4 | 83.4 ± 8.8 | 52.30 | –53.84 | 374 | 3.1 | –1.5 | 0.0 |
| SFA6 | 2023-02-26 | Winter | Ovigerous | 11 | 9811 | 10.6 ± 2.1 | 24.4 ± 1.4 | 105.1 ± 7.8 | 51.63 | –52.05 | 392 | 3.8 | –0.9 | 0.0 |
| SFA6 | 2023-02-26 | Winter | Males | 9 | 9801 | 4.8 ± 1.0 | 19.9 ± 1.6 | 85.3 ± 9.8 | 51.63 | –52.05 | 392 | 3.8 | –0.9 | 0.0 |
| SFA6 | 2023-04-20 | Spring | Ovigerous | 10 | 9811 | 11.0 ± 1.7 | 25.5 ± 1.4 | 107.4 ± 9.4 | 52.37 | –53.82 | 376 | 3.4 | –0.6 | 28.7 |
| SFA6 | 2023-04-20 | Spring | Females | 2 | 9810 | 7.3 ± 0.5 | 23.2 ± 0.8 | 102.1 ± 5.5 | 52.37 | –53.82 | 376 | 3.4 | –0.6 | 28.7 |
| SFA6 | 2023-04-20 | Spring | Males | 9 | 9801 | 4.3 ± 0.4 | 19.2 ± 0.7 | 85.7 ± 4.2 | 52.37 | –53.82 | 376 | 3.4 | –0.6 | 28.7 |
| SFA2 | 2023/07/18 | Summer | Zooplankton | 3 | N/A | N/A | N/A | N/A | 56.28 | –59.75 | 529 | 3.0 | 7.1 | 69.2 |
| SFA2 | 2023/07/20 | Summer | Zooplankton | 3 | N/A | N/A | N/A | N/A | 57.71 | –60.20 | 219 | 1.1 | 5.9 | 68.0 |
| SFA4 | 2023/07/23 | Summer | Zooplankton | 3 | N/A | N/A | N/A | N/A | 58.72 | –61.19 | 203 | 0.8 | 5.6 | 75.6 |
| SFA4 | 2023/07/23 | Summer | Zooplankton | 3 | N/A | N/A | N/A | N/A | 59.38 | –60.32 | 435 | 4.2 | 4.5 | 47.8 |
| SFA4 | 2023/07/25 | Summer | Zooplankton | 3 | N/A | N/A | N/A | N/A | 60.50 | –61.23 | 759 | 4.1 | 4.0 | 38.6 |
| SFA4 | 2023/07/26 | Summer | Zooplankton | 3 | N/A | N/A | N/A | N/A | 60.44 | –62.58 | 333 | 2.0 | 4.0 | 85.1 |
| SFA5 | 2023/07/27 | Summer | Zooplankton | 3 | N/A | N/A | N/A | N/A | 60.73 | –64.33 | 349 | 1.3 | 3.7 | 78.6 |
| SFA5 | 2023/07/28 | Summer | Zooplankton | 3 | N/A | N/A | N/A | N/A | 61.43 | –63.25 | 643 | 4.0 | 3.5 | 82.8 |

**^a^** Sampling date (year/month/day).

**^b^** Number of total individuals per station and maturity stage used for trophic marker analyses.

**^c^** Sexual maturity stages codes: **9801** - Male; **9806** - Female (1^st^-time spawner) with head roe; **9809** - Female (multiple spawner) with head roe; **9810** - Female (multiple spawner) no head roe; **9811** - Ovigerous Female.

^d^SIC: Average of sea ice concentration three months before sampling.
