# Supplementary material for: Trophic ecology and nutritional status of northern shrimp in Canada’s sub-Arctic
Source: PLoS One. 2025 May 20;20(5):e0322745. doi: 10.1371/journal.pone.0322745 (PMC12091755; doi:10.1371/journal.pone.0322745)
Supplement: S2 Table — (DOCX) [file pone.0322745.s004.docx]

**S2 Table.** Isotopic composition of northern shrimp (*Pandalus borealis*) in Canada’s sub-Arctic regions.

| **SFA^a^** | **Total Weight (wet/frozen)**  **(mg)** | **Carapace Length**  **(mm)** | **Total Length (mm)** | **n^b^** | *δ***^15^N (‰)^c^** | *δ***^13^C (‰)^d^** |  | **Total Weight (wet/frozen)**  **(mg)** | **Carapace Length (mm)** | **Total Length (mm)** | **n^b^** | *δ***^15^N (‰)^c^** | *δ***^13^C (‰)^d^** |
| --- | --- | --- | --- | --- | --- | --- | --- | --- | --- | --- | --- | --- | --- |
|  | **Females** | | | | | |  | **Males** | | | | | |
| SFA2 | 11.0 ± 1.9 | 26.2 ± 1.6 | 109.7 ± 10.7 | 46 | 11.0 ± 0.5 | –18.9 ± 0.7 |  | 5.8 ± 1.8 | 21.5 ± 2.4 | 93.0 ± 10.5 | 28 | 10.8 ± 0.4 | –19.1 ± 0.7 |
| SFA3 | 8.7 ± 1.5 | 23.2 ± 1.7 | 101.6 ± 8.7 | 39 | 11.7 ± 0.4 | –17.4 ± 0.6 |  | 3.9 ± 0.7 | 18.3 ± 1.5 | 81.1 ± 7.0 | 32 | 11.1 ± 0.5 | –18.0 ± 0.4 |
| SFA4 | 9.5 ± 1.7 | 25.0 ± 1.9 | 10.6 ± 9.2 | 75 | 10.7 ± 0.4 | –18.7 ± 0.5 |  | 5.2 ± 1.3 | 20.5 ± 2.4 | 89.0 ± 8.6 | 84 | 10.4 ± 0.3 | –18.5 ± 0.5 |
| SFA5 | 9.3 ± 1.8 | 24.0 ± 1.7 | 103.1 ± 9.8 | 50 | 10.9 ± 0.4 | –18.4 ± 0.3 |  | 4.3 ± 1.6 | 18.6 ± 2.4 | 84.7 ± 11.8 | 47 | 10.5 ± 0.4 | –18.4 ± 0.4 |
| SFA6 | 10.2 ± 1.7 | 24.3 ± 1.7 | 107.0 ± 10.6 | 38 | 10.8 ± 0.4 | –18.6 ± 0.4 |  | 4.4 ± 1.0 | 19.0 ± 1.6 | 85.6 ± 7.8 | 34 | 10.5 ± 0.4 | –18.8 ± 0.5 |

^a^ SFA = Shrimp Fishing Area

^b^ Number of total individuals per species used for stable isotope analyses.

^c^ Mean values ± standard deviation of δ^15^N (‰).

^d^ Mean values ± standard deviation of δ^13^C (‰).
