# Supplementary material for: Trophic ecology and nutritional status of northern shrimp in Canada’s sub-Arctic
Source: PLoS One. 2025 May 20;20(5):e0322745. doi: 10.1371/journal.pone.0322745 (PMC12091755; doi:10.1371/journal.pone.0322745)
Supplement: S3 Table — (DOCX) [file pone.0322745.s005.docx]

**S3 Table.** Summary of the main effects on stable isotope (*δ*^13^C – *δ*^15^N) values of northern shrimp (*Pandalus borealis*) across five shrimp fishing areas in Canada’s sub-Arctic regions.

| **Model** | **Main effects and significant interaction effects** | **Estimate** | **Lower.CL**  **2.5%** | **Upper.CL**  **97.5%** | **p-value** |
| --- | --- | --- | --- | --- | --- |
| ***δ*^13^C** | **Shrimp fishing areas (SFAs)** |  |  |  |  |
| ANOVA | SFA2 - SFA3 | –1.292 | –1.527 | –1.056 | < 0.001*** |
|  | SFA2 - SFA4 | –0.389 | –0.589 | –0.189 | < 0.001*** |
|  | SFA2 - SFA5 | –0.531 | –0.750 | –0.311 | < 0.001*** |
|  | SFA3 - SFA4 | 0.903 | 0.700 | 1.106 | < 0.001*** |
|  | SFA3 - SFA5 | 0.761 | 0.539 | 0.983 | < 0.001*** |
|  | SFA3 - SFA6 | 1.054 | 0.817 | 1.292 | < 0.001*** |
|  | SFA5 - SFA6 | 0.294 | 0.073 | 0.514 | 0.003** |
| Linear Model | **Maturity stages, weight and length** | | | | |
|  | Females - Males | –0.639 | –0.830 | –0.448 | < 0.001*** |
|  | Ms - caparace (mm) | –0.077 | –0.120 | –0.034 | < 0.001*** |
|  | **Environmental variables** |  |  |  |  |
|  | Sea Ice (%) | –0.003 | –0.004 | –0.001 | 0.007** |
|  | Surface temperature | 0.046 | 0.020 | 0.073 | 0.001*** |
|  | Bottom temperature | –0.218 | –0.250 | –0.185 | < 0.001*** |
| ***δ*^15^N** | **Shrimp fishing areas (SFAs)** | | | | |
| ANOVA | SFA2 - SFA3 | –0.556 | –0.763 | –0.349 | < 0.001*** |
|  | SFA2 - SFA4 | 0.319 | 0.143 | –0.494 | < 0.001*** |
|  | SFA2 - SFA6 | 0.252 | 0.046 | –0.459 | 0.008** |
|  | SFA3 - SFA4 | 0.875 | 0.696 | 1.053 | < 0.001*** |
|  | SFA3 - SFA5 | 0.745 | 0.550 | 0.940 | < 0.001*** |
|  | SFA3 - SFA6 | 0.808 | 0.599 | 1.017 | < 0.001*** |
|  | **Seasons** | | | | |
|  | Autumn - Spring | 0.447 | 0.285 | 0.609 | < 0.001*** |
|  | Autumn - Summer | 0.590 | 0.430 | 0.750 | < 0.001*** |
|  | Autumn - Winter | 0.412 | 0.260 | 0.565 | < 0.001*** |
|  | Summer - Winter | –0.177 | –0.354 | –0.001 | 0.048* |
| Linear Model | **Maturity stages, weight and length** | | | | |
|  | Females - Males | –0.342 | –0.503 | –0.181 | < 0.001*** |
|  | **Environmental variables** | | | | |
|  | Bottom depth | 0.002 | 0.001 | 0.002 | < 0.001*** |
|  | Sea Ice (%) | –0.005 | –0.006 | –0.003 | < 0.001*** |
|  | Bottom temperature | –0.089 | –0.119 | –0.060 | < 0.001*** |

Confidence level used: 0.95

The level of statistical significance: ***p *<* 0.001, **p *<* 0.01, *p *<* 0.05.
