# Supplementary material for: Trophic ecology and nutritional status of northern shrimp in Canada’s sub-Arctic
Source: PLoS One. 2025 May 20;20(5):e0322745. doi: 10.1371/journal.pone.0322745 (PMC12091755; doi:10.1371/journal.pone.0322745)
Supplement: S4 Table — (DOCX) [file pone.0322745.s006.docx]

**S4 Table.** Summary of the main effects on saturated, monounsaturated and polyunsaturated fatty acid values of northern shrimp (*Pandalus borealis*) across five shrimp fishing areas in Canada’s sub-Arctic regions.

| **Model** | **Main effects and significant interaction effects** | **Estimate** | **Lower.CL**  **2.5%** | **Upper.CL**  **97.5%** | **p-value** |
| --- | --- | --- | --- | --- | --- |
| **Saturates** | **Shrimp fishing areas (SFAs)** | | | | |
| ANOVA | SFA2 - SFA3 | –2.067 | –3.063 | –1.071 | < 0.001*** |
|  | SFA2 - SFA4 | –2.536 | –3.419 | –1.652 | < 0.001*** |
|  | SFA2 - SFA5 | –2.518 | –3.469 | –1.566 | < 0.001*** |
|  | SFA3 - SFA6 | 1.359 | 0.255 | 2.464 | 0.007** |
|  | SFA4 - SFA6 | 1.828 | 0.824 | 2.832 | < 0.001*** |
|  | SFA5 - SFA6 | 1.810 | 0.745 | 2.875 | < 0.001*** |
|  | **Seasons** | | | | |
|  | Autumn - Spring | 1.323 | 0.434 | 2.211 | < 0.001*** |
|  | Spring - Summer | –1.603 | –2.563 | –0.644 | < 0.001*** |
|  | Spring - Winter | –1.690 | –2.658 | –0.721 | < 0.001*** |
| Linear Model | **Maturity stages (Ms), weight and length** | | | | |
|  | Females - Males | –2.177 | –3.008 | –1.345 | < 0.001*** |
|  | Ms - weight (gr) | –0.493 | –0.631 | –0.354 | < 0.001*** |
|  | **Environmental variables** | | | | |
|  | Bottom depth | –0.007 | –0.012 | –0.003 | 0.001*** |
|  | Bottom temperature | –0.346 | –0.487 | –0.205 | < 0.001*** |
| **Monounsaturates** | **Shrimp fishing areas (SFAs)** | | | | |
| ANOVA | SFA2 - SFA4 | 4.085 | 1.40 | 6.768 | < 0.001*** |
|  | SFA2 - SFA5 | 4.389 | 1.50 | 7.279 | < 0.001*** |
|  | SFA2 - SFA6 | –3.319 | –6.56 | –0.074 | 0.042* |
|  | SFA3 - SFA4 | 5.199 | 2.38 | 8.015 | < 0.001*** |
|  | SFA3 - SFA5 | 5.502 | 2.49 | 8.516 | < 0.001*** |
|  | SFA4 - SFA6 | –7.404 | –10.45 | –4.353 | < 0.001*** |
|  | SFA5 - SFA6 | –7.707 | –10.94 | –4.473 | < 0.001*** |
| Linear Model | **Maturity stages, weight and length** |  |  |  |  |
|  | Eggs - Females | –4.926 | –7.88 | –1.97 | < 0.001*** |
|  | Eggs - Males | –5.552 | –8.53 | –2.58 | < 0.001*** |
|  | Females - Males | 5.261 | 2.685 | 7.837 | < 0.001*** |
|  | Females + Males - weight (gr) | 0.939 | 0.511 | 1.367 | < 0.001*** |
|  | **Environmental variables** | | | | |
|  | Bottom depth | 0.022 | 0.008 | 0.035 | 0.002** |
| **Polyunsaturates** | **Shrimp fishing areas (SFAs)** | | | | |
| ANOVA | SFA2 - SFA3 | 3.422 | 1.01 | 5.832 | 0.001*** |
|  | SFA2 - SFA6 | 4.123 | 1.54 | 6.709 | < 0.001*** |
|  | SFA3 - SFA4 | –4.781 | –7.02 | –2.537 | < 0.001*** |
|  | SFA3 - SFA5 | –4.837 | –7.24 | –2.435 | < 0.001*** |
|  | SFA4 - SFA6 | 5.483 | 3.05 | 7.913 | < 0.001*** |
|  | SFA5 - SFA6 | 5.538 | 2.96 | 8.115 | < 0.001*** |
| Linear Model | **Maturity stages (Ms), weight and length** | | | | |
|  | Eggs - Females | 6.537 | 4.232 | 8.84 | < 0.001*** |
|  | Eggs - Males | 7.298 | 4.979 | 9.62 | < 0.001*** |
|  | Females - Males | –2.709 | –4.736 | –0.682 | 0.009** |
|  | Females + Males - weight (gr) | –0.394 | –0.731 | –0.057 | 0.042* |
|  | **Environmental variables** | | | | |
|  | Bottom depth | –0.015 | –0.026 | –0.004 | 0.008** |

Confidence level used: 0.95

The level of statistical significance: ***p *<* 0.001, **p *<* 0.01, *p *<* 0.05.
