# Supplementary material for: Trophic ecology and nutritional status of northern shrimp in Canada’s sub-Arctic
Source: PLoS One. 2025 May 20;20(5):e0322745. doi: 10.1371/journal.pone.0322745 (PMC12091755; doi:10.1371/journal.pone.0322745)
Supplement: S5 Table — (DOCX) [file pone.0322745.s007.docx]

**S5 Table.** Summary of the main effects on lipid classes and total lipid content values of northern shrimp (*Pandalus borealis*) across five shrimp fishing areas in Canada’s sub-Arctic regions.

| **Model** | **Main effects and significant interaction effects** | **Estimate** | **Lower.CL**  **2.5%** | **Upper.CL**  **97.5%** | **p-value** |
| --- | --- | --- | --- | --- | --- |
| **Triacylglycerols** | **Seasons** | | | | |
| ANOVA | Autumn - Summer | 23.13 | 13.85 | 32.409 | < 0.001*** |
|  | Autumn - Winter | –10.32 | –19.65 | –0.985 | 0.024* |
|  | Spring - Summer | 18.99 | 7.40 | 30.590 | < 0.001*** |
|  | Spring - Winter | –14.46 | –26.10 | –2.815 | 0.008** |
|  | Summer - Winter | –33.45 | –44.18 | –22.720 | < 0.001*** |
| Linear Model | **Maturity stages (Ms), weight and length** | | | | |
|  | Females - Males | 28.150 | 14.742 | 41.557 | < 0.001*** |
|  | Ms - weight (gr) | 4.990 | 2.911 | 7.069 | < 0.001*** |
|  | **Environmental variables** | | | | |
|  | Sea Ice (%) | –0.312 | –0.422 | –0.202 | < 0.001*** |
|  | Surface temperature | –2.491 | –3.894 | –1.087 | 0.001*** |
| **Phospholipids** | **Seasons** | | | | |
| ANOVA | Autumn - Summer | –0.475 | –0.894 | –0.057 | 0.019** |
|  | Summer - Winter | –0.859 | 0.375 | 1.343 | < 0.001*** |
| Linear Model | **Maturity stages, weight and length** |  |  |  |  |
|  | Eggs - Males | 0.913 | 0.602 | 1.224 | < 0.001*** |
|  | Eggs - Females | 0.998 | 0.616 | 1.380 | < 0.001*** |
|  | Females - Males | –1.154 | –1.642 | –0.665 | < 0.001*** |
|  | Ms - weight (gr) | –0.315 | –0.459 | –0.172 | < 0.001*** |
|  | Ms - caparace (mm) | 0.169 | 0.047 | 0.290 | 0.007** |
|  | **Environmental variables** |  |  |  |  |
|  | Sea Ice (%) | 0.005 | 0.001 | 0.010 | 0.031* |
|  | Surface temperature | 0.070 | 0.006 | 0.134 | 0.031* |
| **Total lipids** | **Seasons** | | | | |
| ANOVA | Autumn - Spring | 0.579 | 0.158 | 0.999 | 0.002** |
|  | Autumn - Summer | 0.567 | 0.189 | 0.945 | < 0.001*** |
|  | Autumn - Winter | –0.386 | –0.766 | –0.005 | 0.045* |
|  | Spring - Winter | –0.964 | –1.438 | –0.490 | < 0.001*** |
|  | Summer - Winter | –0.952 | –1.389 | –0.515 | < 0.001*** |
| Linear Model | **Maturity stages (Ms), weight and length** | | | | |
|  | Eggs - Males | 1.22 | 0.955 | 1.489 | < 0.001*** |
|  | Eggs - Females | 1.02 | 0.694 | 1.350 | < 0.001*** |
|  | Females - Males | 0.667 | 0.228 | 1.105 | 0.003** |
|  | Ms - length (mm) | 0.018 | 0.001 | 0.036 | 0.042* |
|  | Ms - weight (gr) | 0.114 | 0.047 | 0.181 | 0.001*** |
|  | **Environmental variables** | | | | |
|  | Sea Ice (%) | –0.020 | –0.024 | –0.015 | < 0.001*** |
|  | Bottom temperature | 0.076 | 0.012 | 0.139 | 0.019* |

Confidence level used: 0.95

The level of statistical significance: ***p *<* 0.001, **p *<* 0.01, *p *<* 0.05.
