# Supplementary material for: Trophic ecology and nutritional status of northern shrimp in Canada’s sub-Arctic
Source: PLoS One. 2025 May 20;20(5):e0322745. doi: 10.1371/journal.pone.0322745 (PMC12091755; doi:10.1371/journal.pone.0322745)
Supplement: S6 Table — Zooplankton samples were collected south to north from various stations including Sentinel, Isecold-1, Isecold-2, SagBank, Hatton Basin, Isecold-3, Killinek Main, and Hatton 600. EPA: polyunsaturated eicosapentaenoic acid; DHA: polyunsaturated docosahexaenoic acid; SFA: saturated FA; MUFA: monounsaturated FA; PUFA: polyunsaturated FA; ω3: Omega-3; ω6: Omega-6, and EFA: essential fatty acids (20:4ω-6; 20:5ω-3; 22:6ω-3). Only those fatty acids ≥1% are included in this table. (DOCX) [file pone.0322745.s008.docx]

**S6 Table.** **Relative abundance of individual fatty acids as a percentage of total FAs (mean values with standard deviations) in zooplankton in Canada’s sub-Arctic regions.** Zooplankton samples were collected south to north from various stations, including Sentinel, Isecold-1, Isecold-2, SagBank, Hatton Basin, Isecold-3, Killinek Main, and Hatton 600. EPA: polyunsaturated eicosapentaenoic acid; DHA: polyunsaturated docosahexaenoic acid; SFA: saturated FA; MUFA: monounsaturated FA; PUFA: polyunsaturated FA; ω3: Omega-3; ω6: Omega-6, and EFA: essential fatty acids (20:4ω-6; 20:5ω-3; 22:6ω-3). Only those fatty acids ≥1% are included in this table.

| **Fatty acids/**  **Station** | **Sentinel**  (n=3) | **Isecold-1**  (n=3) | **Isecold-2**  (n=3) | **SagBank**  (n=3) | **Hatton Basin**  (n=3) | **Isecold-3**  (n=3) | **Killinek Main**  (n=3) | **Hatton 600**  (n=3) | **Whole**  **stations** |
| --- | --- | --- | --- | --- | --- | --- | --- | --- | --- |
| **Saturates (%)** | | | | | | | | | |
| 14:0 | 4.6 ± 0.3 | 5.9 ± 0.3 | 4.9 ± 0.7 | 5.8 ± 0.3 | 7.6 ± 0.4 | 5.4 ± 0.3 | 4.6 ± 0.5 | 4.9 ± 0.4 | 5.4 ± 1.0 |
| i16:0 | 2.9 ± 0.5 | 1.9 ± 0.7 | 1.8 ± 0.7 | 3.1 ± 0.6 | 4.0 ± 0.2 | 4.0 ± 0.2 | 3.8 ± 0.9 | 3.8 ± 0.5 | 3.2 ± 1.0 |
| 16:0 | 5.3 ± 0.3 | 6.0 ± 0.3 | 7.8 ± 0.8 | 5.5 ± 0.4 | 7.6 ± 0.4 | 9.9 ± 1.2 | 6.4 ± 0.6 | 5.2 ± 1.2 | 6.7 ± 1.7 |
| **ΣSFA** | 11.4 ± 0.6 | 13.4 ± 0.5 | 13.9 ± 1.6 | 12.8 ± 0.7 | 17.2 ± 0.7 | 17.5 ± 1.1 | 13.8 ± 1.6 | 11.9 ± 1.8 | 14.0 ± 2.4 |
| **Monounsaturates (%)** | | | | | | | | | |
| 16:1ω7 | 15.9 ± 0.6 | 16.8 ± 0.3 | 22.0 ± 1.7 | 14.3 ± 0.3 | 11.9 ± 0.1 | 22.2 ± 1.2 | 18.3 ± 0.5 | 13.7 ± 0.3 | 16.9 ± 3.7 |
| 18:1ω7 | 1.1 ± 0.1 | 0.9 ± 0.1 | 1.9 ± 0.1 | 0.8 ± 0.03 | 1.1 ± 0.1 | 1.9 ± 0.2 | 1.3 ± 0.1 | 1.1 ± 0.2 | 1.3 ± 0.4 |
| 18:1ω9 | 2.6 ± 0.1 | 3.2 ± 0.2 | 3.2 ± 0.3 | 5.2 ± 0.3 | 7.1 ± 0.3 | 8.0 ± 0.7 | 5.2 ± 0.1 | 4.0 ± 0.7 | 4.8 ± 1.9 |
| 20:1ω9 | 7.1 ± 0.3 | 7.8 ± 0.7 | 5.7 ± 0.5 | 6.5 ± 0.2 | 6.3 ± 0.2 | 5.1 ± 0.5 | 7.6 ± 0.6 | 7.0 ± 0.5 | 6.6 ± 1.0 |
| 22:1ω-11 | 6.8 ± 0.2 | 6.5 ± 0.8 | 5.1 ± 0.5 | 6.5 ± 0.02 | 5.7 ± 0.2 | 3.5 ± 0.2 | 5.9 ± 0.5 | 6.1 ± 0.2 | 5.7 ± 1.1 |
| **ΣMUFA** | 37.9 ± 1.1 | 40.3 ± 2.3 | 41.8 ± 3.0 | 37.7 ± 0.5 | 36.7 ± 0.6 | 44.5 ± 1.6 | 42.1 ± 1.2 | 36.6 ± 0.3 | 39.7 ± 3.1 |
| **Polyunsaturates (%)** | | | | | | | | | |
| 16:3ω4 | 1.5 ± 0.02 | 1.0 ± 0.04 | 1.3 ± 0.1 | 0.9 ± 0.003 | 0.6 ± 0.01 | 1.0 ± 0.1 | 1.2 ± 0.1 | 0.8 ± 0.04 | 1.0 ± 0.3 |
| 16:4ω1 | 3.4 ± 0.1 | 2.7 ± 0.1 | 2.4 ± 0.1 | 2.5 ± 0.1 | 1.1 ± 0.02 | 2.4 ± 0.1 | 3.0 ± 0.1 | 1.8 ± 0.1 | 2.4 ± 0.7 |
| 18:2ω6 (LIN) | 0.9 ± 0.03 | 1.1 ± 0.03 | 0.8 ± 0.1 | 1.2 ± 0.04 | 1.2 ± 0.04 | 1.6 ± 0.2 | 1.3 ± 0.1 | 1.1 ± 0.04 | 1.1 ± 0.3 |
| 18:4ω3 | 4.0 ± 0.1 | 3.9 ± 0.2 | 2.6 ± 0.1 | 4.6 ± 0.1 | 4.5 ± 0.04 | 2.1 ± 0.3 | 2.4 ± 0.1 | 4.4 ± 0.2 | 3.6 ± 1.0 |
| 20:5ω3 (EPA) | 27.8 ± 1.0 | 25.8 ± 2.3 | 25.2 ± 2.7 | 27.2 ± 0.2 | 23.6 ± 0.2 | 18.1 ± 1.3 | 23.1 ± 1.5 | 27.1 ± 1.5 | 24.7 ± 3.3 |
| 22:5ω3 | 2.1 ± 0.02 | 2.5 ± 0.2 | 1.6 ± 0.3 | 2.1 ± 0.2 | 1.5 ± 0.04 | 1.0 ± 0.1 | 1.8 ± 0.03 | 2.8 ± 0.4 | 2.0 ± 0.6 |
| 22:6ω3 (DHA) | 3.8 ± 0.1 | 3.2 ± 0.3 | 4.5 ± 0.5 | 3.6 ± 0.3 | 5.4 ± 0.1 | 3.0 ± 0.6 | 2.6 ± 1.2 | 4.9 ± 0.3 | 3.9 ± 1.0 |
| **ΣPUFA** | 47.4 ± 1.2 | 43.8 ± 2.0 | 41.8 ± 3.8 | 45.8 ± 0.4 | 41.2 ± 0.9 | 33.1 ± 2.2 | 39.9 ± 1.0 | 47.0 ± 2.3 | 42.5 ± 4.8 |
| **Bacterial** | 4.5 ± 0.4 | 3.3 ± 0.6 | 3.3. ± 0.9 | 4.6 ± 0.6 | 5.8 ± 0.3 | 5.9 ± 0.4 | 5.3 ± 0.8 | 5.6 ± 0.8 | 4.8 ± 1.1 |
| **P/S** | 4.2 ± 0.3 | 3.3 ± 0.3 | 3.1 ± 0.7 | 3.6 ± 0.2 | 2.4 ± 0.1 | 1.9 ± 0.2 | 2.9 ± 0.4 | 4.0 ± 0.7 | 3.2 ± 0.8 |
| **Σω3** | 39.4 ± 1.1 | 37.0 ± 2.1 | 35.3 ± 3.8 | 39.3 ± 0.4 | 36.7 ± 0.6 | 25.8 ± 2.3 | 31.7 ± 1.9 | 41.2 ± 1.9 | 35.8 ± 5.1 |
| **Σω6** | 2.9 ± 0.1 | 2.8 ± 0.1 | 2.7 ± 0.1 | 2.9 ± 0.1 | 2.7 ± 0.2 | 3.9 ± 0.3 | 3.7 ± 0.5 | 3.0 ± 0.3 | 3.1 ± 0.5 |
| **Σω6/Σω3** | 0.1 ± 0.003 | 0.1 ± 0.01 | 0.1 ± 0.01 | 0.1 ± 0.004 | 0.1 ± 0.005 | 0.2 ± 0.02 | 0.1 ± 0.02 | 0.1 ± 0.003 | 0.1 ± 0.03 |
| **DHA/EPA** | 0.1 ± 0.004 | 0.1 ± 0.02 | 0.2 ± 0.01 | 0.1 ± 0.01 | 0.2 ± 0.01 | 0.2 ± 0.02 | 0.1 ± 0.05 | 0.2 ± 0.02 | 0.2 ± 0.04 |
| **Zooplankton** | 16.3 ± 0.5 | 17.6 ± 1.7 | 13.0 ± 1.1 | 15.6 ± 0.2 | 14.3 ± 0.4 | 10.2 ± 1.0 | 15.6 ± 1.1 | 16.0 ± 0.9 | 14.8 ± 2.3 |
| **Vascular plants** | 1.3 ± 0.1 | 1.4 ± 0.03 | 1.0 ± 0.1 | 1.7 ± 0.05 | 1.6 ± 0.4 | 1.9 ± 0.2 | 1.5 ± 0.1 | 1.6 ± 0.04 | 1.5 ± 0.3 |
| **ΣEFA** | 31.8 ± 1.1 | 29.1 ± 2.1 | 29.9 ± 3.2 | 30.9 ± 0.1 | 29.1 ± 0.2 | 21.3 ± 2.0 | 25.7 ± 2.8 | 32.2 ± 1.4 | 28.8 ± 3.8 |
