# Supplementary material for: Trophic ecology and nutritional status of northern shrimp in Canada’s sub-Arctic
Source: PLoS One. 2025 May 20;20(5):e0322745. doi: 10.1371/journal.pone.0322745 (PMC12091755; doi:10.1371/journal.pone.0322745)
Supplement: S7 Table — The average lipid class contents (as mg/g WW) are measured in milligrams per gram of wet weight. (DOCX) [file pone.0322745.s009.docx]

**S7 Table. The relative concentration of lipid classes in zooplankton in Canada’s sub-Arctic regions.** The average lipid class contents (as mg/g WW) are measured in milligrams per gram of wet weight.

| **Station** | **n^a^** | **Hydrocarbons** | **Steryl Esters/**  **Wax Esters** | **Ethyl Esters/**  **Methyl Esters** | **TAG^b^** | **FFA^c^** | **Alcohols** | **Sterols** | **Acetone Mobile** | **PL^d^** | **Amount Extracted** |
| --- | --- | --- | --- | --- | --- | --- | --- | --- | --- | --- | --- |
| Sentinel | 3 | 0.3 ± 0.1 | 13.9 ± 8.2 | 7.9 ± 4.4 | 1.5 ± 0.6 | 0.6 ± 0.2 | 0.2 ± 0.03 | 0.2 ± 0.1 | 0.2 ± 0.04 | 0.2 ± 0.2 | 25.0 ± 13.6 |
| Isecold-1 | 3 | 0.2 ± 0.02 | 4.5 ± 0.4 | 2.5 ± 0.1 | 0.7 ± 0.1 | 0.3 ± 0.1 | 0.1 ± 0.1 | 0.03 ± 0.01 | 0.04 ± 0.01 | 0.2 ± 0.3 | 8.5 ± 0.4 |
| Isecold-2 | 3 | 0.1 ± 0.04 | 3.5 ± 1.5 | 2.3 ± 1.1 | 2.3 ± 0.9 | 0.6 ± 0.1 | 0.1 ± 0.1 | 0.2 ± 0.1 | 0.1 ± 0.1 | 0.3 ± 0.3 | 9.6 ± 4.0 |
| SagBank | 3 | 0.3 ± 0.1 | 11.2 ± 3.4 | 6.7 ± 2.2 | 1.5 ± 0.8 | 0.6 ± 0.2 | 0.2 ± 0.02 | 0.1 ± 0.1 | 0.2 ± 0.1 | 1.0 ± 1.5 | 21.7 ± 8.5 |
| Hatton Basin | 3 | 0.2 ± 0.03 | 13.4 ± 2.0 | 8.2 ± 1.4 | 2.0 ± 0.7 | 0.5 ± 0.1 | 0.2 ± 0.01 | 0.2 ± 0.1 | 0.1 ± 0.1 | 5.6 ± 9.0 | 30.5 ± 12.6 |
| Isecold-3 | 3 | 0.03 ± 0.03 | 0.8 ± 0.5 | 0.4 ± 0.3 | 0.4 ± 0.2 | 0.2 ± 0.2 | 0.002 ± 0.003 | 0.01 ± 0.01 | 0.1 ± 0.1 | 0.6 ± 0.7 | 2.6 ± 1.6 |
| Killinek Main | 3 | 0.2 ± 0.03 | 9.0 ± 0.1 | 2.9 ± 0.2 | 2.0 ± 0.2 | 0.5 ± 0.04 | 0.1 ± 0.01 | 0.2 ± 0.01 | 0.2 ± 0.01 | 0.3 ± 0.2 | 15.3 ± 0.2 |
| Hatton 600 | 3 | 0.4 ± 0.1 | 15.7 ± 5.9 | 9.3 ± 3.1 | 0.9 ± 0.5 | 0.6 ± 0.1 | 0.1 ± 0.1 | 0.1 ± 0.1 | 0.3 ± 0.04 | 0.3 ± 0.3 | 27.5 ± 8.9 |

^a^ Number of total individuals per type used for lipid classes analysis.

^b^ TAG: Triacylglycerols

^c^ FFA: Free Fatty Acids

^d^ PL: Phospholipids
