# Supplementary material for: Trophic ecology and nutritional status of northern shrimp in Canada’s sub-Arctic
Source: PLoS One. 2025 May 20;20(5):e0322745. doi: 10.1371/journal.pone.0322745 (PMC12091755; doi:10.1371/journal.pone.0322745)
Supplement: S8 Table — (DOCX) [file pone.0322745.s010.docx]

**S8 Table. Isotopic composition of zooplankton across Canada’s sub-Arctic regions.**

| **Station** | **Date Sampling^a^** | **n^b^** | ***δ*^15^N (‰)^c^** | ***δ*^13^C (‰)^d^** | **%N** | **%C** | **C/N (mg/mg)** |
| --- | --- | --- | --- | --- | --- | --- | --- |
| Sentinel | 2023-07-18 | 3 | 7.7 ± 0.3 | –24.3 ± 0.1 | 7.0 ± 0.2 | 49.3 ± 1.9 | 7.0 ± 0.2 |
| Isecold-1 | 2023-07-20 | 3 | 6.3 ± 0.1 | –23.6 ± 0.1 | 6.5 ± 0.1 | 42.5 ± 1.4 | 6.5 ± 0.3 |
| Isecold-2 | 2023-07-23 | 3 | 7.0 ± 0.1 | –22.7 ± 0.1 | 8.8 ± 0.1 | 49.1 ± 0.2 | 5.6 ± 0.1 |
| SagBank | 2023-07-23 | 3 | 7.6 ± 0.3 | –24.2 ± 0.2 | 7.7 ± 0.3 | 49.5 ± 1.5 | 6.5 ± 0.3 |
| Hatton Basin | 2023-07-25 | 3 | 7.2 ± 0.1 | –23.6 ± 0.3 | 8.7 ± 0.2 | 48.1 ± 0.1 | 5.5 ± 0.1 |
| Isecold-3 | 2023-07-26 | 3 | 7.0 ± 0.1 | –22.0 ± 0.2 | 6.3 ± 0.2 | 33.5 ± 0.6 | 5.3 ± 0.2 |
| Killinek Main | 2023-07-27 | 3 | 7.9 ± 0.2 | –22.4 ± 0.1 | 7.7 ± 0.1 | 53.0 ± 0.3 | 6.9 ± 0.04 |
| Hatton 600 | 2023-07-28 | 3 | 7.7 ± 0.01 | –24.7 ± 0.1 | 8.1 ± 0.2 | 51.4 ± 2.7 | 6.3 ± 0.3 |

^a^ Sampling date (year/month/day).

^b^ Number of total individuals per species used for stable isotope analyses.

^c^ Mean values ± standard deviation of δ^15^N (‰).

^d^ Mean values ± standard deviation of δ^13^C (‰).
